# Multi‐omics consensus ensemble refines the classification of muscle‐invasive bladder cancer with stratified prognosis, tumour microenvironment and distinct sensitivity to frontline therapies

**DOI:** 10.1002/ctm2.601

**Published:** 2021-12-22

**Authors:** Xiaofan Lu, Jialin Meng, Liwen Su, Liyun Jiang, Haitao Wang, Junkai Zhu, Mengjia Huang, Wenxuan Cheng, Li Xu, Xinjia Ruan, Shuyuan Yeh, Chaozhao Liang, Fangrong Yan

**Affiliations:** ^1^ State Key Laboratory of Natural Medicines Research Center of Biostatistics and Computational Pharmacy China Pharmaceutical University Nanjing P.R. China; ^2^ Department of Urology The First Affiliated Hospital of Anhui Medical University Institute of Urology Anhui Province Key Laboratory of Genitourinary Diseases Anhui Medical University Hefei Anhui P.R. China; ^3^ Department of Biostatistics The University of Texas MD Anderson Cancer Center Texas USA; ^4^ Cancer Center Faculty of Health Sciences Center for Precision Medicine Research and Training University of Macau Macau P.R. China; ^5^ George Whipple Lab for Cancer Research Departments of Pathology Urology, Radiation Oncology and The Wilmot Cancer Institute University of Rochester Medical Center Rochester New York USA; ^6^ Present address: Center for Cancer Research Clinical Research/NCI/NIH Bethesda MD 20892 USA

Dear Editor,

The molecular classification of muscle‐invasive bladder cancer (MIBC) based on transcriptomic signatures has been extensively studied.^1^, ^2^, ^3^, ^4^ The complementary nature of information provided by different molecular profiles motivated us to refine MIBC classification by aggregating multi‐omics data. The entire workflow is outlined in Figure 1A, information on the eight datasets is summarized in Tables S1‐S2, and technical details are listed in the Supporting Materials and Methods.

**FIGURE 1:**
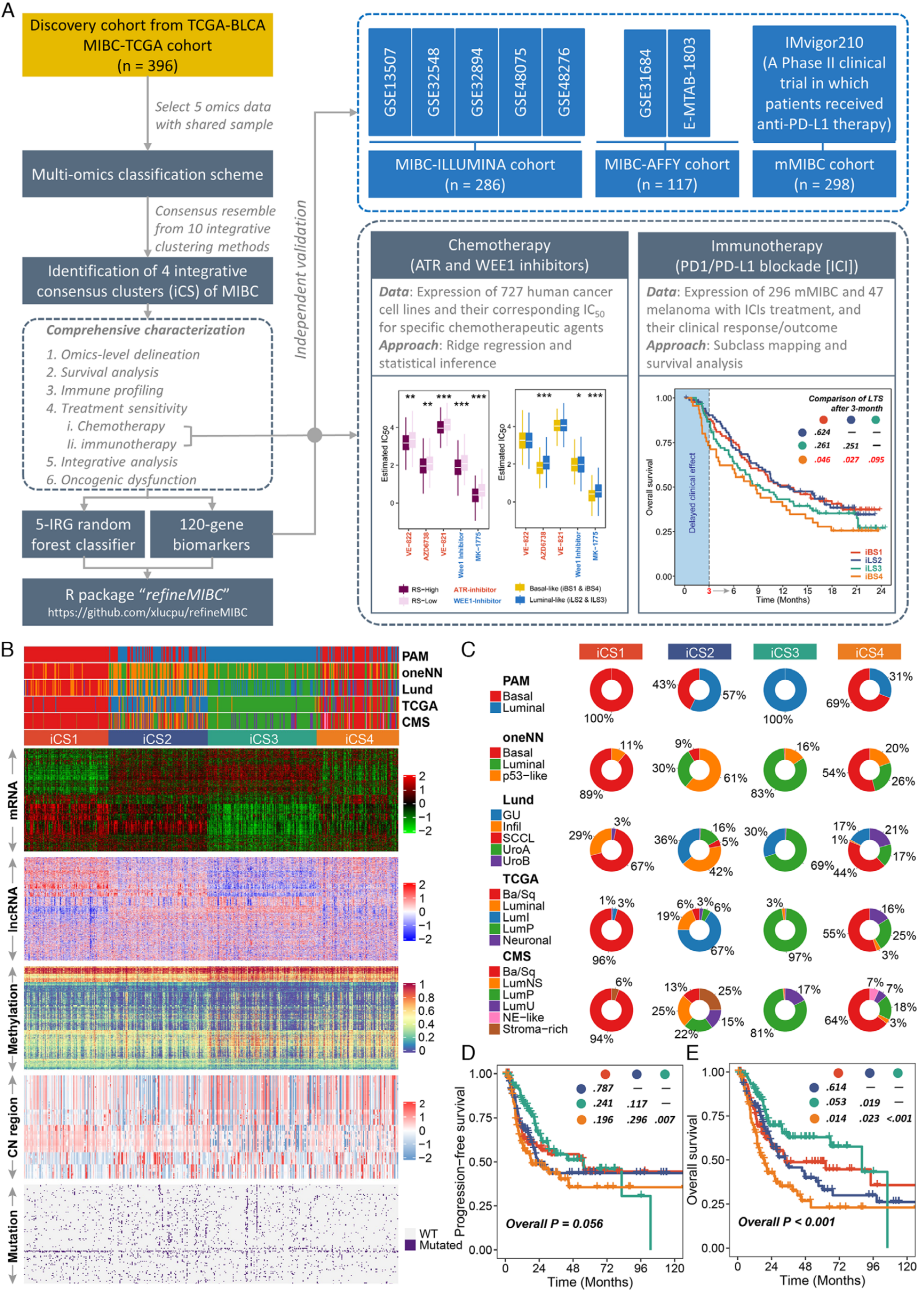
The multi‐omics consensus ensemble identifies four molecular subtypes of MIBC. (A) Design overview. This study enrolled a total of 1097 muscle‐invasive bladder cancer (MIBC) cases and identified four MIBC subtypes under multi‐omics framework that stratify prognosis, tumour microenvironment and distinct sensitivity to frontline therapies. An R package '*refineMIBC*' was provided for MIBC refinement in clinical setting. (B) Comprehensive heatmap showing the molecular landscape of four integrative consensus subtypes (iCSs) of MIBC (*n* = 396). Other previously defined gene expression‐based MIBC subtypes were annotated at the top of the heatmap, including prediction analysis of microarrays‐based (PAM), one nearest neighbour (oneNN) prediction model‐based, Lund, TCGA and consensus molecular subtypes (CMS). (C) Pie charts showing the proportion of other gene expression‐based MIBC subtypes in the current iCS. Kaplan–Meier curves of (D) progression‐free survival and (E) overall survival with log‐rank test for 396 MIBC patients stratified by iCS

A consensus ensemble was generated through 10 multi‐omics integrative clustering approaches on five omics datasets of 396 MIBCs from the TCGA database.^5^ We identified four robust integrative consensus subtypes (iCSs; Figure S1A,B), which showed distinctive molecular patterns and were significantly associated with clinicopathological features and previously identified molecular classifications (Figure 1B); iCS1 and iCS4 dramatically overlapped with basal‐like subtypes, whereas iCS2 and iCS3 were enriched for luminal‐like subtypes (*p *< 0.001; Figure 1C, Table S3). We relabelled iCS1 to iCS4 as iBS1, iLS2, iLS3, and iBS4, respectively. Our classification system was tightly associated with prognosis (Figure 1D,E), showing superior performance than PAM, oneNN, and Lund classifications, but inferior than TCGA and consensus molecular subtype (CMS) classifications regarding overall stratification (Figure S2); iBS4 with the worst outcome that was refined from basal‐like subtypes caught our attention.

Regulon analysis strongly manifested the biological pertinency of a four‐classification where differentiated activity of cancerous chromatin remodelling regulons highlighted other possible differential regulatory patterns among iCSs (Figure 2A), indicating that epigenetically driven transcriptional networks might be important differentiators of these subtypes.

**FIGURE 2:**
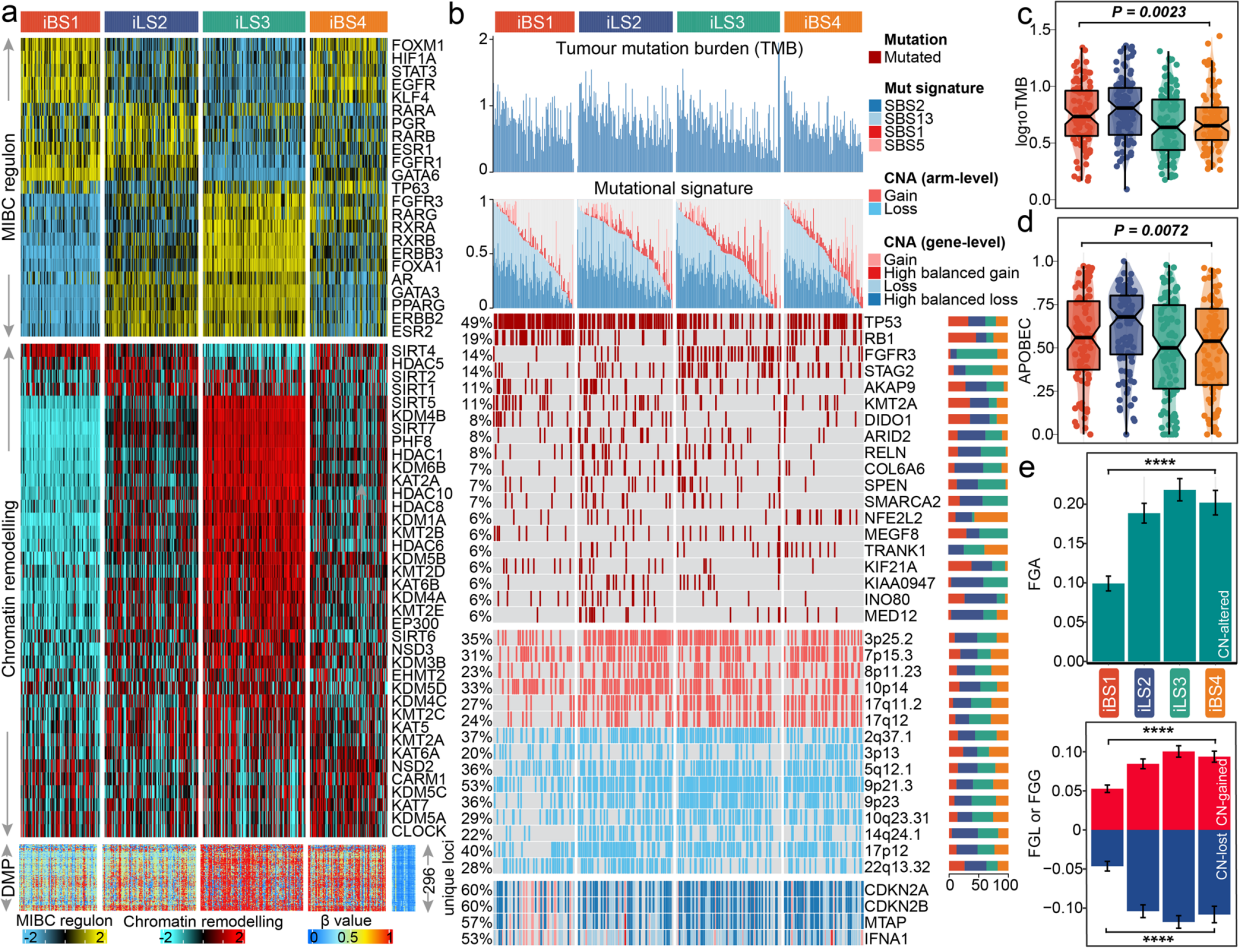
Molecular landscape of four MIBC iCSs. (A) Heatmap showing profiles of regulon activity for 23 regulator (top panel), and potential regulators that are relevant with chromatin remodelling (middle panel); Similar patterns were shared by iBS1 and iBS4, but iBS1 differed with high activity of *GATA6, FGFR1* and *ESR1* for regulon activity, while iBS4 was distinctly associated with high activity of *TP63*. A total of 296 unique differentially methylated promoters derived from each iCS versus adjacent normal samples (bottom panel); the iLS3 (265 vs. 45 in iLS2) and iBS4 (191 vs. 26 in iBS1) had more hypermethylated promoters (296 unique loci) than the 21 normal samples had. (B) Genomic alteration landscape according to iCS. Samples are sorted in descending order according to the cumulative contribution of APOBEC‐relevant mutational signatures (i.e., SBS2 + SBS13) within each iCS. TMB, relative contribution of four mutational signatures, selected differentially mutated genes (>5%) and broad‐level CNAs (>20%), and selected genes located within Chr9p21.3 are shown from the top to the bottom panels. Of note, iBS1 harboured more mutations of *TP53* (74%; *p *< 0.001), *RB1* (39%; *p *< 0.001) and *KMT2A* (18%; *p *= 0.033) than others, while iBS4 was enriched in mutations of *NFE2L2* (16%; *p *= 0.001; *p *= 0.005 compared to iBS1 [3%]) and *TRANK1* (10%; *p *= 0.06; *p *= 0.001 compared to iBS1 [0%]); *KIAA0947* (11%; *p *= 0.005), *MED12* (11%; *p *= 0.005), *COL6A6* (13%; *p *= 0.008), and *ARID2* (14%; *p *= 0.01) were mutated more frequently in iLS2, whereas iLS3 was enriched in mutations of *FGFR3* (34%; *p *< 0.001), *STAG2* (22%; *p *= 0.006), and *SPEN* (11%; *p *= 0.05). The proportion of iCS in each alteration is presented in the right bar charts. The distributions of TMB and APOBEC contributions are shown in (C) and (D), respectively. (E) Distribution of fraction genome altered (FGA) and fraction genome gain/loss (FGA/FGG). Bar charts are presented as the mean ± standard error of the mean. Statistical *p* values were calculated by Kruskal–Wallis rank sum test for multiple comparison

We observed that iLS2 showed a higher tumour mutation burden (TMB, *p *= 0.002; Figure 2B,C) and harboured more mutations in APOBEC‐relevant signatures (*p *= 0.007; Figure 2D). Of the frequently mutated genes (>5%), iBS1 harboured more mutations of *TP53*, *RB1* and *KMT2A*, while iBS4 was enriched for mutations in *NFE2L2* (also known as *NRF2*) and *TRANK1*; *KIAA0947*, *MED12*, *COL6A6*, and *ARID2* were mutated more frequently in iLS2, whereas iLS3 was enriched for mutations in *FGFR3*, *STAG2* and *SPEN* (Table S4). iBS1 had better chromosomal stability with lower copy number alterations (CNAs) (*p *< 0.001; Figure 2E). Ch9p21.3 was susceptible to inactivation in cell immortalization and diseases; within this region, interferon‐alpha (*IFNA*) genes, *MTAP* and *CDKN2A/B* were differentially altered in iBS1 compared to others, which may contribute to shaping different basal‐like subtypes (Figure 2B).

We generated a 120‐gene signature to predict iCSs in external cohorts with 403 MIBCs (Figures S3A,B and S4A‐C, Table S5). The signature‐predicted iCSs highly overlapped with the CMS but further refined the basal‐like classification (*p *< 0.001; Figure S4D,E). Consistently, iBS4 showed the most unfavourable prognosis (Figure S4F‐G).

Since cancer immunity plays a critical role in tumour progression, tumour microenvironment deconvolution suggested that immunocyte infiltration was dramatically higher in both iBS1 and iLS2 than in the other subtypes (Figure 3A, Table S6); iBS1 had relatively higher expression of genes that represent potential targets for immunotherapy. The immune landscape was validated (Figure S5A,B). Additionally, we found a similar immunologic profile from the IMvigor210 cohort (*n* = 298), in which the clinical effect of PD‐L1 blockade with atezolizumab was evaluated in metastatic MIBC patients (Figure 3B), and a higher proportion of iBS1 achieved a complete response (*p *= 0.024; Figure 3C). The predicted iBS4 showed poorer outcome than immune‐hot iCSs (i.e., iBS1 and iLS2; *p *< 0.05 at 6 months, *p *< 0.1 at 12 months) and had a segregated survival curve compared to immune‐cold iLS3 (Figure S6A). Due to the potentially delayed clinical effect of immunotherapy, we also compared the long‐term overall survival (OS) rates after three months of treatment, leading to the observation that iBS4 was associated with poorer long‐term OS than iBS1 (*p *= 0.046), iLS2 (*p *= 0.027) and iLS3 (*p *= 0.095), suggesting its high malignancy and potential resistance to immune checkpoint inhibitors (ICIs) (Figure S6B).

**FIGURE 3:**
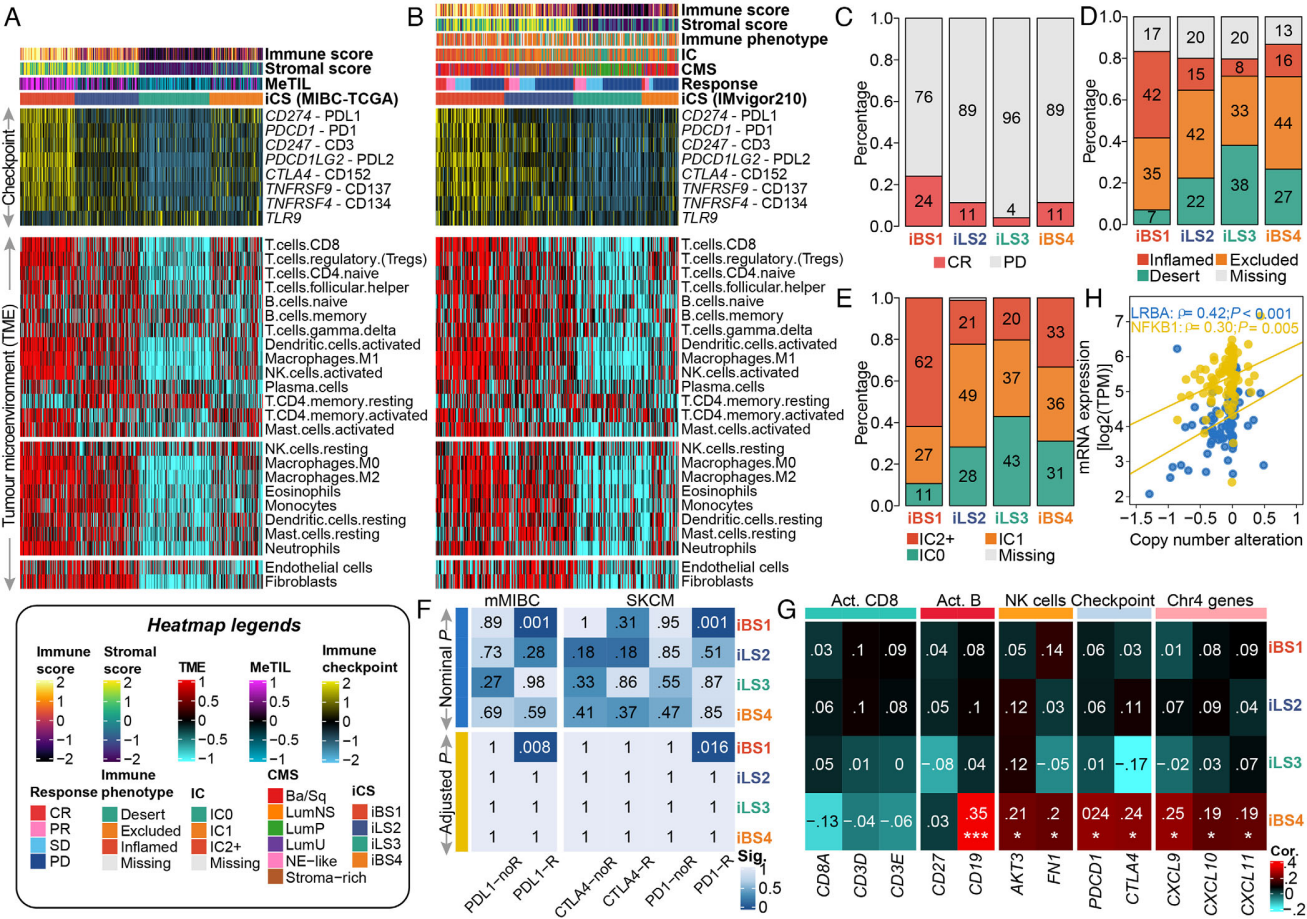
Differential immune profile across MIBC subtypes and its association with genomic alteration and immunotherapeutic response. (A) Heatmap showing the immune profile in the MIBC‐TCGA cohort, with the top panel showing the expression of genes involved in immune checkpoint targets and the bottom panel showing the enrichment level of 24 microenvironment cell types. The immune enrichment score, stromal enrichment score and DNA methylation of tumour‐infiltrating lymphocytes (MeTILs) were annotated at the top of the heatmap. (B) Immune profile heatmap for the IMvigor210 cohort with annotations for immune enrichment score, stromal enrichment score, immune phenotype, immune cell (IC) level, and best confirmed overall response, including four categories, namely, complete response (CR), partial response (PR), stable disease (SD), and progressive disease (PD). Barplots showing the distribution of (C) CR and PD, (D) immune phenotype and (E) IC levels in iCS of the IMvigor210 cohort. (F) Subclass analysis revealed that the iBS1 subtype could be more sensitive to the anti‐PD‐L1 and anti‐PD1 agents (both, Bonferroni‐corrected *p *= 0.001) using two reference cohorts in which patients received immunotherapy. (G) Heatmap showing the correlation between Chr4 copy number deletion and expression profiles of immune markers across ICSs in the MIBC‐TCGA cohort. A positive correlation indicates that the deletion of Chr4 may trigger the downregulation of the relevant immune gene expression. (H) Positive correlation between downregulation of expression of immune regulatory genes and Chr4 deletion in immune‐cold iBS4 of the MIBC‐TCGA cohort; statistical *p* value was calculated by Spearman rank‐order correlation coefficient

Mariathasan et al. reported that the clinical effect of anti‐PD‐L1 blockade may be influenced by tumour immune and immune cell phenotypes.^6^ In this context, we renamed iBS1 ‘basal‐inflamed', iBS4 ‘basal‐noninflamed', iLS2 ‘luminal‐excluded', and iLS3 ‘luminal‐desert' according to the phenotype proportions (Figure 3D,E). SubMap revealed that only the basal‐inflamed subtype showed a high likelihood of responding to ICIs (FDR < 0.01; Figures 3F and S7), which indicated that the current classification may be useful to identify ideal candidates for immunotherapy, especially for basal‐like MIBC.

Recently, Hao et al. suggested that Chr4 loss could induce an unfavourable ‘immune‐cold' phenotype.^7^ We demonstrated that only the basal‐noninflamed subtype showed significant Chr4p and 4q deletions (FDR < 0.25; Table S7), and Chr4p loss was tightly linked to unfavourable OS (HR = 1.56, 95% CI: 1.001–2.437, *p *= 0.0496). Chr4 harbours several immune regulatory genes and genes that encode chemokines which are crucial for T cell recruitment. Additionally, Chr4 deletion is genetically linked with immune deficiency syndromes.^8^ Integrative analysis of RNA‐Seq and CNA indicated that Chr4 deletion in the basal‐noninflamed subtype may drive the decreased expression of key immune markers and their key mediator, *NFKB1* (Figure 3G‐H).

Enrichment analysis revealed that the cell cycle oncogenic pathway was activated in basal‐inflamed/noninflamed MIBC (*p *< 0.001; Figures 4A,B and S8A,B). Activation of the cell cycle pathway induces cell cycle checkpoint regulatory proteins (e.g., ataxia telengiectasia and Rad3‐related [ATR] and WEE1) involved in replication stress, which are associated with DNA damage responses that contribute to cisplatin resistance; such an association mirrored a potentially higher sensitivity of responding to ATR and WEE1 inhibitors in basal‐inflamed/noninflamed MIBC (Figures 4D,E and S8C,D). A recent study reported that *NRF2* enables an immune‐cold microenvironment by inducing *COX2*/*PGE2* and inhibiting the DNA‐sensing innate immune response^9^; consistently, the luminal‐desert/basal‐noninflamed subtypes with activated *NRF2* oncogenic pathway had dramatically higher *COX2* expression levels than others in both the TCGA and IMvigor210 (Figures 4F and S9A‐C) cohorts, suggesting that dysfunction of oncogenic pathways might drive the low immune infiltration of these MIBCs. Unfortunately, it is not feasible to combine the currently unavailable *NRF2* inhibitors with ICIs. Nevertheless, preclinical models have demonstrated that *COX* inhibitors and anti‐PD‐1 immunotherapy have a synergistic effect^10^; therefore, targeting *NRF2* downstream markers provides a therapeutic opportunity for immune‐cold MIBCs. Additionally, the different biological characteristics of the four subtypes may indicate the need for different targeted therapeutic interventions (Table S8).

**FIGURE 4:**
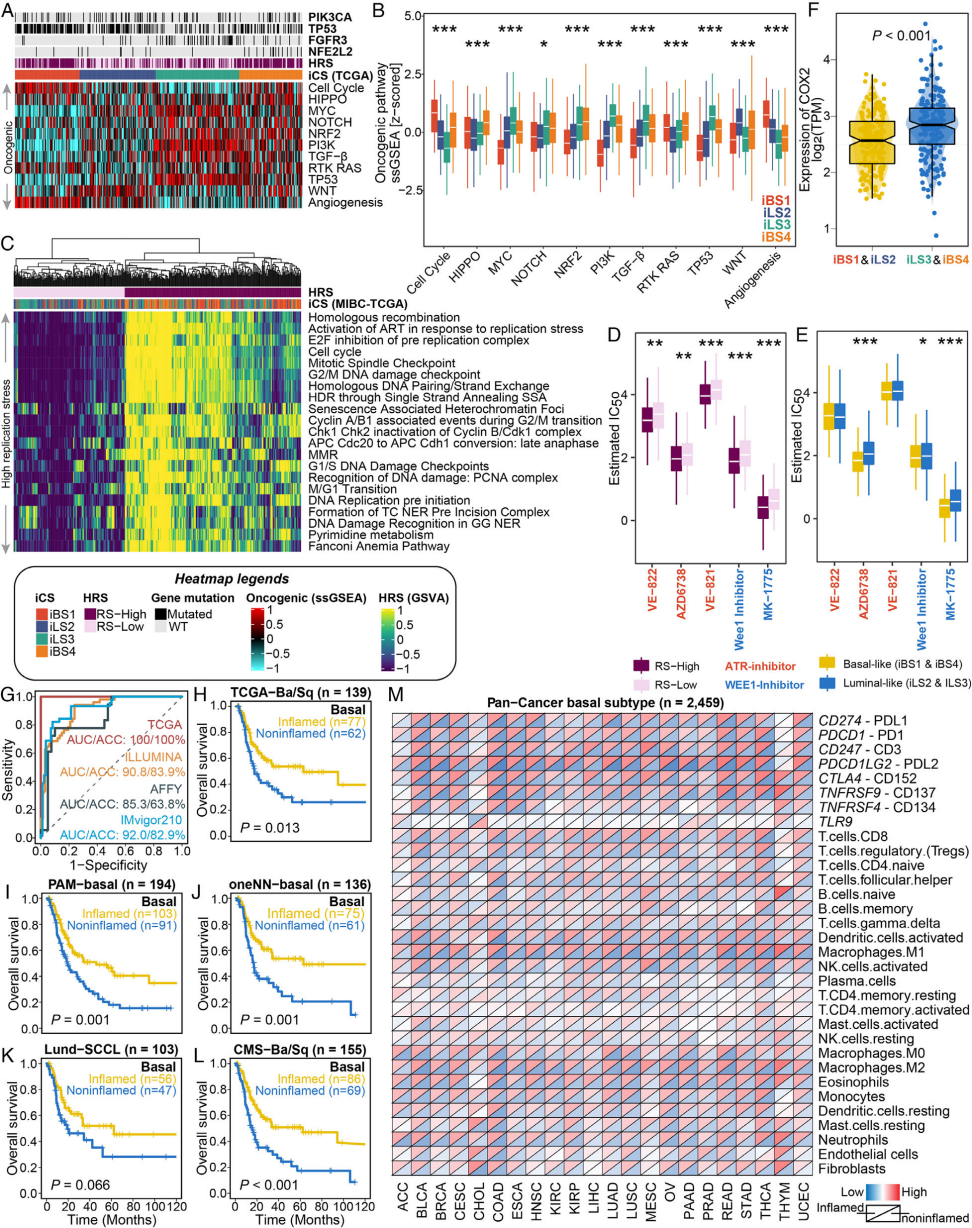
Oncogenic pathways, replication stress and targeted inhibitors in MIBC as well as predictive performance of random forest basal‐classifier and its application in pan‐carcinoma investigation. Dysfunctional oncogenic pathways quantified by single‐sample gene set enrichment analysis are presented in (A) a heatmap and (B) a boxplot for the MIBC‐TCGA cohort. Relevant mutations involved in several oncogenic pathways are annotated at the top. The cell cycle oncogenic pathway was significantly activated in basal‐inflamed/noninflamed MIBC; the luminal‐desert and basal‐noninflamed subtypes showed relatively higher activation of the oncogenic *NRF2* pathway; the luminal‐excluded subtype was highly enriched in the *WNT* pathway, while the luminal‐desert subtype showed the lowest enrichment in angiogenesis genes but the highest activation of the *TGF‐β* pathway. (C) Enrichment heatmap showing the signalling pathways (Reactome database) that are involved in DNA maintenance as well as activated cell cycle regulation in DNA damage response and replication stress. Two replication stress (RS) subtypes were identified for the MIBC‐TCGA cohort. Subtypes with (D) high replication stress (RS‐High) or (E) basal‐like MIBC (iBS and iBS4) were inferred to be much more sensitive to both ATR (i.e., VE‐822, AZD6739 and VE‐821) and WEE1 (i.e., Wee1 inhibitor and MK‐1775) inhibitors by applying a ridge regression model using 727 human cancer cell lines. Drug sensitivity was measured as ln(IC_50_), and the lower the value was, the more sensitive the patient would be to the treatment. (F) Distribution of *COX2* expression between immune‐hot (i.e., iBS1 and iLS2) and immune‐cold (i.e., iLS2 and iBS4) phenotypes of MIBC‐TCGA. (G) ROC curve showing predictive performance (area under the curve [AUC] and accuracy [ACC]) when using the basal‐classifier to refine basal‐like MIBC into basal‐inflamed and basal‐noninflamed subtypes. The prognostic value of the basal‐classifier in refining previously identified basal‐like subtypes of MIBC is presented in (H) for the TCGA‐basal/squamous subtype, (I) for the PAM‐basal subtype, (J) for the oneNN‐basal subtype, (K) for the Lund‐SCCL subtype, and (L) for the CMS‐Ba/Sq subtype. (M) Diagonal heatmap showing global immunological divergence across 22 basal‐like carcinomas with a total of 2459 cases. The upper triangle of each heatmap cell represents the average expression of the immune checkpoint/cell in the predicted basal‐inflamed subtype given a specific tumour type, while the lower triangle represents the predicted basal‐noninflamed subtype

Given the distinct molecular and prognostic characteristics of basal‐inflamed/noninflamed MIBC compared with traditional basal‐like classifications, we developed a random forest predictor, which is superior than decision tree, to refine basal‐like MIBC (Table S9‐10, Figure S10). The final basal classifier contained five immune‐related genes, *C3AR1*, *CCL8*, *FCGR3A*, *LILRB2* and *PDCD1LG2*, and the predictor achieved superior performance (Figure 4G) and showed the capability of prognostic stratification in refining different basal‐like classifications (Figure 4H‐L). Strikingly, almost all kinds of epithelial tumours could be refined into immune‐hot or immune‐cold basal‐like phenotypes (Figure 4M), suggesting a global immunological divergence across basal‐like carcinomas.

We acknowledge the limitations of our study, where signals quantified by bulk RNA‐Seq and microarray profiles were confounded from mixed cell populations; thus, incorporating these findings with multiplex immunohistochemistry assays to investigate intrinsic tumour cell variation and their crosstalk with the tumour microenvironment, which interferes with the therapeutic response is warranted.

In summary, we identified four MIBC subtypes with distinct landscapes using a multi‐omics approach that stratifies prognosis, tumour microenvironment characteristics and distinct sensitivity to frontline therapies. Additionally, we offered the R package “‘*refineMIBC*' as a translational research tool to refine MIBC classification from a single‐sample perspective in retrospective or prospective studies.

## CONFLICT OF INTEREST

The authors have no conflict of interest.

## DATA AVAILABILITY STATEMENT

We developed the R package, “*refineMIBC*”, which is documented and freely available at https://github.com/xlucpu/refineMIBC. This package implements a 120‐gene template that assigns subtype labels according to the multi‐omics consensus ensemble of muscle‐invasive bladder cancer (MIBC) using nearest template prediction. The consensus ensemble identifies 4 integrative consensus subtypes: basal‐inflamed, basal‐noninflamed, luminal‐excluded, and luminal‐desert. This package also deploys a 5‐immune‐gene classifier to refine each basal‐like MIBC as either basal‐inflamed or basal‐noninflamed by a random forest classifier if basal‐like classification has already been identified by other approaches (e.g., CMS, PAM, oneNN, Lund, etc.).

## Supporting information

Supplementary information
