# Supplementary material for: Multi‐omics consensus ensemble refines the classification of muscle‐invasive bladder cancer with stratified prognosis, tumour microenvironment and distinct sensitivity to frontline therapies
Source: Clin Transl Med. 2021 Dec 22;11(12):e601. doi: 10.1002/ctm2.601 (PMC8693439; doi:10.1002/ctm2.601)
Supplement: Supplementary file 1 — Supplementary information [file CTM2-11-e601-s001.docx]

**MULTI-OMICS CONSENSUS ENSEMBLE REFINES THE CLASSIFICATION OF MUSCLE-INVASIVE BLADDER CANCER WITH STRATIFIED PROGNOSIS, TUMOUR MICROENVIRONMENT AND DISTINCT SENSITIVITY TO FRONTLINE THERAPIES**

**INDEX OF SUPPORTING DATA**

**SUPPLEMENTARY MATERIALS AND METHODS ………………………..…. Page 2**

**SUPPLEMENTARY REFERENCES ………………...……………….…………… Page 8**

**SUPPLEMENTARY TABLES S1-10 ..……………………………………….……. Page 12**

**SUPPLEMENTARY FIGURES S1-10 ..…………………………………….……... Page 32**

**SUPPLEMENTARY MATERIALS AND METHODS**

**Multi-omics data sets**

Molecular profiles of the TCGA-BLCA dataset were retrieved as MIBC-TCGA cohort used for the multi-omics data analysis ^1^, including 396 primary muscle-invasive bladder cancers with complete transcriptome expression, somatic mutations, CNAs, DNA methylation and survival outcomes available. For raw count data of high-throughput sequencing downloaded by the R package “*TCGAbiolinks 2.16.3*” ^2^ (parameter list: project = “TCGA-BLCA”, data.category = “Transcriptome Profiling”, data.type = “Gene Expression Quantification”, workflow.type = “HTSeq – Counts”), both mRNAs and lncRNAs were considered. Specifically, lncRNAs were identified according to Vega (<http://vega.archive.ensembl.org/>) for the following types: non_coding, 3prime_overlapping_ncRNA, antisense_RNA, lincRNA, sense_intronic, sense_overlapping, macro_lncRNA, and bidirectional_promoter_lncRNA. Ensembl IDs for transcriptomes were transformed into gene symbols by GENCODE27 mapping. The number of fragments per kilobase million (FPKM) was computed and converted into transcripts per kilobase million (TPM), which showed more similarity to the numbers obtained from microarray analysis and improved comparability between samples. DNA methylation profile was downloaded from the XENA database (<https://xenabrowser.net/>). Copy number segment data were collected from FireBrowse (<http://firebrowse.org/>). Somatic mutations, clinicopathological features, overall survival (OS) and progression-free survival (PFS) rate data were downloaded from cBioPortal (<https://www.cbioportal.org/>).

**External transcriptome data sets**

Seven microarray data sets with transcriptome expression profiles and overall clinical outcomes were used for external validation. Patients with early-stage BCa were defined as samples with a T-stage of Ta or T1, indicating that these tumours were only in the innermost layer of the bladder lining (Ta) or had started to grow into the connective tissue beneath the bladder lining (T1); these tumours were commonly classified as NMIBC and were initially removed for this study. Specifically, five microarray data sets that were quantified by Illumina beadchip were combined as the MIBC-ILLUMINA cohort, including GSE13507 (n = 61) ^3^, GSE32548 (n = 38) ^4^, GSE32894 (n = 51) ^5^, GSE48075 (n = 72) ^6^, GSE48276 (n = 64) ^7^; two microarray data sets that were sequenced by Affymetrix genechip were combined as the MIBC-AFFY cohort with the removal of patients who received chemo/radiotherapies, including GSE31684 (n = 74) ^8^ and E-MTAB-1803 (n = 43) ^9^. Information about the platform and corresponding sample size of the eight MIBC data sets are summarized **in Table S1**; demographic and clinical characteristic descriptions are detailed in **Table S2**. For microarray data, the median value was considered if the gene symbol was annotated with multiple probe IDs. The potential cross-dataset batch effect was removed under an empirical Bayes framework by the R package “*sva 3.36.0*” with parameters by default ^10^, and the batch effect was further investigated using principal component analysis (**Figure S2a-b**).

**Multi-omics integration and visualization**

To perform integrative clustering, the MIBC-TCGA multi-omics data sets were primed to form five data matrixes of which rows correspond to the features and columns correspond to the common samples (n = 396). The transcriptome expression profile was first log_2_ transformed. For the methylation data, we extracted probes located in promoter CpG islands, and the median β value was considered for genes having more than one probe mapping to its promoter, resulting in 10,871 methylated genes. For the mutation matrix, a gene was considered mutated (entry of 1) if it contained at least one type of the following nonsynonymous variations: missense/nonsense/nonstop mutation, frameshift deletion/insertion, in-frame deletion/insertion, translation start site or splice site mutation; otherwise, 0 was used to designate wild-type status. For the CNAs, we condensed the genomic segments as described in the literature ^11^. To better fit the model and accelerate the clustering efficiency, features with flat values were removed. Specifically, we selected the top 1,500 most variable mRNAs, lncRNAs, methylation genes, and copy number regions according to the median absolute deviation. Additionally, genes with mutation rates >3% (n = 1,358) were selected for subtyping.

To find an optimal clustering number, we referred to the number of previous molecular subtypes of MIBC and calculated the clustering prediction index (CPI) and gap statistics ^12^. Consequently, integrative clustering of the MIBC-TCGA cohort was independently conducted by 10 state-of-the-art multi-omics integrative clustering algorithms ^13^. We borrowed the idea of a consensus ensemble for later integration of the clustering results derived from different algorithms to improve the clustering robustness ^14^. To be specific, we defined a consensus matrix, which is an $\left( N\times N \right)$ matrix that stores, for each pair of samples, the proportion of clustering algorithms in which two samples are clustered together. Mathematically, let $M^{\left( x \right)}$ denotes the $\left( N\times N \right)$ connectivity matrix corresponding to integrative clustering algorithm $\left( x \right)$. The entries of this matrix are defined as follows:

$$M^{\left( x \right)}\left( i,j \right)=\left\{ \begin{aligned} 1 if samples i\mathrm{and}j belong to the same cluster. \\ 0 \mathrm{otherwise}. \end{aligned} \right.$$

Finally, the consensus matrix $\mathcal{M}$ can then be defined as sum of the connectivity matrices of all the algorithms divided by the total number of algorithms that used:

$$\mathcal{M}\left( i,j \right)=\frac{\sum_{x} M^{\left( x \right)}\left( i,j \right)}{10}$$

That is, the entry $\left( i,j \right)$ in the consensus matrix records the agreement that how sample $i$ and $j$ are assigned to the same cluster by different algorithms. Notably, the consensus matrix is symmetric, in that $\mathcal{M}\left( i,j \right)=\mathcal{M}\left( j,i \right)$, and each entry is a real number range from 0 to 1. Besides, we defined $1-\mathcal{M}$ as a new distance measurement and conducted hierarchical tree construction to define the final clusters.

**Calculation of microenvironment cell abundance and pathway enrichment**

To establish a compendium of gene list related to specific microenvironment cells, two gene signatures (CIBERSORT ^15^ and MCPcounter ^16^) were modified. As CIBERSORT does not contain signatures related to fibroblasts and endothelial cells, extra 40 genes were added to account for these cells (32 genes for endothelial cells and 8 genes for fibroblasts) from MCPcounter to our compendium, which consisted of 364 genes representing 24 microenvironment cell types. We then used gene set variation analysis (GSVA) on these gene sets to generate enrichment scores for each cell using the R package “*GSVA 1.36.2*” with parameters by default. The presence of infiltrating immune/stromal cells in the tumour tissue was estimated by the R package “*estimate 1.0.13*” with parameters by default ^17^. Furthermore, the score of DNA methylation of tumour-infiltrating lymphocyte (MeTIL) in the MIBC-TCGA cohort was calculated individually according to the protocols outlined in the literature ^18^. We referred to a published paper and the angiogenesis gene set (<https://www.gsea-msigdb.org/gsea/msigdb/cards/ANGIOGENESIS>) to construct a signature of ten oncogenic pathways, and the GSVA method was harnessed to generate enrichment scores ^19^. A total of 21 replication stress signatures were retrieved from the literature, and single-sample gene set enrichment analysis (ssGSEA) was performed to quantify the enrichment level ^20^. Two subtypes of replication stress were identified by hierarchical clustering.

**Bioinformatic analyses**

We analysed the mutation landscape by the R package “*maftools 2.4.12*” with the initial removal of 100 FLAGS and other parameters by default ^21^, and we evaluated the mutational signatures through the R package “*deconstructSigs 1.9.0*” with parameters by default ^22^. Four mutational signatures showed a high correlation with bladder cancer were inferred, namely, SBS1 (age-related), SBS2 and SBS13 (APOBEC activity-related) and SBS5 (*ERCC2* mutation-related). Recurrent focal somatic CNAs were detected and localized by GISTIC2.0 through GenePattern (<https://www.genepattern.org/>), with the thresholds of copy number amplifications/deletions being equal to ± 0.3 (q-value < 0.05) ^23^. The individual fraction of copy number-altered genome (FGA) for the MIBC-TCGA cohort was calculated based on copy number segment data as follows:

$$R={copy number of segments}/2$$

$$FGA={B_{r}}/B$$

The FGA is the fraction of the genome with an value of log_2_(copy number) larger than 0.3 versus the genome with copy number profiled where $B_{r}$ denotes the number of bases in segments with $\left| {log}_{2}R \right|>0.3$ and $B$ represents the number of bases in all segments ^12^. Differential methylation analysis was conducted on probes located in promoter CpG islands between tumour and adjacent normal samples by the R package “*ChAMP 2.18.2*” with parameters by default ^24^; hypermethylated promoters were determined according to the following stringent criteria: the mean methylation β value in the tumour samples was greater than 0.5 and less than 0.2 in the adjacent normal samples with FDR < 0.05. In addition to Lund ^5^, one nearest neighbor (oneNN) prediction model-based ^7^ and prediction analysis of microarray-based (PAM) ^25^ subtypes that were previously identified for MIBC-TCGA, we used the R package “*consensusMIBC 1.1.0*” with parameters by default to broadly predict individual consensus molecular subtypes (CMSs) for each MIBC, including basal/squamous (Ba/Sq), luminal papillary (LumP), luminal unstable (LumU), luminal non-specified (Luminal), neuroendocrine-like (NE-like), and stroma-rich subtypes ^26^. Each sample in the external cohorts was further classified as one of the identified iCSs by nearest template prediction (NTP) ^27^.

**Regulon analysis**

As previously described ^1^, we used the R package “*RTN 2.12.1*” to reconstruct transcriptional regulatory networks (regulons) including a total of 23 ‘regulator’ genes that were associated induced/repressed targets: the steroid hormone receptors *ESR1/2*, *AR* and *PGR*; the nuclear receptors *PPARG*, three *RARs* (*A*/*B*/*G*), and three *RXRs* (*A*/*B*/*G*); the receptor tyrosine kinases *ERBB2*/*3* and *FGFR1*/*3*; and the transcription factors *FOXA1*, *FOXM1*, *GATA3*/*6*, *HIF1A*, *KLF4* and *STAT3* and *TP63*; another panel of 71 candidate regulators that were relevant to cancerous chromatin remodelling was also investigated ^28^. Specifically, mutual information analysis and Spearman rank-order correlation deduced the possible associations between a regulator and all potential target from the transcriptome expression profile, and permutation analysis (n=1,000) was utilized to erase associations with an FDR > 0.00001. Bootstrapping strategy removed unstable associations through one thousand times of resampling with consensus bootstrap greater than 95%. Data processing inequality filtering eliminated the weakest associations in triangles of two regulators and common targets; additional targets were removed from large unbalanced regulons when the subset of either positive or negative targets is below the ‘minRegulonSize’ of 15. Individual regulon activity was estimated by two-sided GSEA.

**Therapeutic response analyses**

Based on the drug sensitivity and phenotype data from GDSC 2016 (<https://www.cancerrxgene.org/>), the R package “*pRRophetic 0.5.0*” was employed with parameters by default to predict the chemotherapeutic sensitivity for each MIBC sample using the expression profiles of 727 human cancer cell lines (CCLs) as the training cohort; the IC_50_ (lower IC_50_ indicates increased sensitivity to treatment) of each sample treated with a specific chemotherapeutic agent was estimated by ridge regression, and 10-fold cross-validation was used to measure the prediction accuracy ^29, 30^. To test the potential therapeutic efficiency of cell cycle checkpoint inhibitors, we estimated the IC_50_ for three ATR inhibitors (*i.e.*, VE-821, VE-822 and AZD6738) and two WEE1 inhibitors (*i.e.*, Wee1 inhibitor and MK-1775) available from GDSC (Version: 2016) through ridge predictors. For immunotherapy, we harnessed subclass mapping (SubMap) to infer the clinical response to immune checkpoint inhibitors ^31^. Specifically, SubMap is an unsupervised method, which estimates the significance of an association (similarity) between subclasses observed in two independent datasets, and the statistical significance of association between subclasses was estimated by using Fisher inverse chi-square statistic. In this manner, we first retrieved a published data set consisting of 47 melanoma patients who responded to immunotherapies ^32^. We then downloaded the IMvigor210 transcriptome profile (n = 298), which originated from a phase II trial that investigated the clinical activity of atezolizumab (anti-PD-L1 agent) for locally advanced and metastatic urothelial carcinoma (mMIBC) ^33^. We calculated the transcriptome TPM values and extracted the best confirmed overall response information using the R package “*IMvigor210CoreBiologies 1.0.0*” ^34^. We collated a list of immune-related genes (IRGs) from the nCounter PanCancer Immune Profiling Panel that consisted of 770 unique genes closely associated with the human immune response ^35^; transcriptome expression of 754 matched IRGs was extracted for subclass analysis.

**Statistical analyses**

All statistical tests were conducted by R 4.0.2, including two-sample Mann-Whitney test for continuous data, Fisher’s exact test for categorical data, log-rank test for Kaplan-Meier curves, and Cox proportional hazards regression for estimating the hazard ratios (HRs) and 95% confidence interval (CI). Non-proportional hazards are observed frequently in cancer immunotherapy trials due to the long-term survival and delayed clinical effect, therefore, the treatment effect of immune checkpoint inhibitors was measured by two non-proportional hazards statistical approaches, namely, restricted mean survival (RMS) and long-term survival inference after three months of treatment, by using the R packages “*survRM2 1.0.3*” and “*ComparisonSurv 1.0.9*”, respectively ^36^. A random forest (RF) predictive model was developed by the R package “*varSelRF 0.7.8*” (parameter list: ntree = 5000, ntreeIterat = 2000, vars.drop.frac = 0.2, whole.range = FALSE, keep.forest = TRUE), technical details of which have been described previously ^37^. To be specific, the MIBC-TCGA cohort was taken as training cohort to construct the predictive model, and other cohorts (*i.e.*, MIBC-ILLUMINA, MIBC-AFFY and IMvigor210) were used as external validation for evaluating the model performance. Next, since distinct landscape of tumor immune microenvironment was observed among basal-like MIBC, differential expression analysis between basal-inflamed and basal-noninflamed MIBC was performed using the R package “*limma 3.44.3*” on ~700 IRGs to select informative immune genes (the top 50 IRGs ordered by log_2_FoldChange for each subtype with FDR<0.05) which served as input into RF model ^38^. For the RF analysis, the input expression matrix was first log_2_-transformed and z-scored, and a backward elimination procedure was then applied to find the optimal biomarkers. Specifically, using the out-of-bag (OOB) error as a minimization criterion, we carried out variable elimination by setting the dropping fraction of each iteration at 0.2, which meant that at each iteration 20% of genes from the bottom of the gene importance ranking list were removed until the OOB error rate reached to its minimum. The group of immune genes when RF reached the smallest OOB error rate was selected as the optimal biomarkers and was used to develop the final RF prediction model, containing five IRGs, *C3AR1*, *CCL8*, *FCGR3A*, *LILRB2*, and *PDCD1LG2*. The receiver operating characteristic (ROC) curves and predictive accuracy (general cutoff of 0.5) were used to assess the model predictive performance. Most of the above analytic processes are embedded in the R package “*MOVICS 0.99.17*”, which we recently developed for multi-omics integration and visualization ^12^. Heatmap illustration was created by R package “*ComplexHeatmap 2.5.5*” ^39^. For all unadjusted comparisons, a two-tailed *P* < 0.05 was considered statistically significant.

**SUPPLEMENTARY REFERENCES**

1. Robertson AG, Kim J, Alahmadie H, Bellmunt J, Guo G, Cherniack AD*, et al.* Comprehensive Molecular Characterization of Muscle-Invasive Bladder Cancer. *Cell* 2017.

2. Colaprico A, Silva TC, Olsen C, Garofano L, Cava C, Garolini D*, et al.* TCGAbiolinks: an R/Bioconductor package for integrative analysis of TCGA data. *Nucleic acids research* 2015, **44**(8)**:** e71-e71.

3. Lee J-S, Leem S-H, Lee S-Y, Kim S-C, Park E-S, Kim S-B*, et al.* Expression signature of E2F1 and its associated genes predict superficial to invasive progression of bladder tumors. *Journal of clinical oncology* 2010, **28**(16)**:** 2660-2667.

4. Lindgren D, Sjödahl G, Lauss M, Staaf J, Chebil G, Lövgren K*, et al.* Integrated genomic and gene expression profiling identifies two major genomic circuits in urothelial carcinoma. *PLoS One* 2012, **7**(6)**:** e38863.

5. Sjödahl G, Lauss M, Lövgren K, Chebil G, Gudjonsson S, Veerla S*, et al.* A molecular taxonomy for urothelial carcinoma. *Clinical cancer research* 2012, **18**(12)**:** 3377-3386.

6. Guo CC, Bondaruk J, Yao H, Wang Z, Zhang L, Lee S*, et al.* Assessment of luminal and basal phenotypes in bladder cancer. *Scientific reports* 2020, **10**(1)**:** 1-14.

7. Choi W, Porten S, Kim S, Willis D, Plimack ER, Hoffman-Censits J*, et al.* Identification of distinct basal and luminal subtypes of muscle-invasive bladder cancer with different sensitivities to frontline chemotherapy. *Cancer cell* 2014, **25**(2)**:** 152-165.

8. Riester M, Taylor JM, Feifer A, Koppie T, Rosenberg JE, Downey RJ*, et al.* Combination of a novel gene expression signature with a clinical nomogram improves the prediction of survival in high-risk bladder cancer. *Clinical cancer research* 2012, **18**(5)**:** 1323-1333.

9. El Behi M, Krumeich S, Lodillinsky C, Kamoun A, Tibaldi L, Sugano G*, et al.* An essential role for decorin in bladder cancer invasiveness. *EMBO Mol Med* 2013, **5**(12)**:** 1835-1851.

10. Leek JT, Johnson WE, Parker HS, Jaffe AE, Storey JD. The sva package for removing batch effects and other unwanted variation in high-throughput experiments. *Bioinformatics* 2012, **28**(6)**:** 882-883.

11. Mo Q, Wang S, Seshan VE, Olshen AB, Schultz N, Sander C*, et al.* Pattern discovery and cancer gene identification in integrated cancer genomic data. *Proc Natl Acad Sci U S A* 2013, **110**(11)**:** 4245-4250.

12. Lu X, Meng J, Zhou Y, Jiang L, Yan F. MOVICS: an R package for multi-omics integration and visualization in cancer subtyping. *Bioinformatics* 2020.

13. Pierre-Jean M, Deleuze J-F, Le Floch E, Mauger F. Clustering and variable selection evaluation of 13 unsupervised methods for multi-omics data integration. *Briefings in Bioinformatics* 2019.

14. Strehl A, Ghosh J. Cluster ensembles---a knowledge reuse framework for combining multiple partitions. *Journal of machine learning research* 2002, **3**(Dec)**:** 583-617.

15. Newman AM, Liu CL, Green MR, Gentles AJ, Feng W, Xu Y*, et al.* Robust enumeration of cell subsets from tissue expression profiles. *Nature methods* 2015, **12**(5)**:** 453.

16. Becht E, Giraldo NA, Lacroix L, Buttard B, Elarouci N, Petitprez F*, et al.* Estimating the population abundance of tissue-infiltrating immune and stromal cell populations using gene expression. *Genome biology* 2016, **17**(1)**:** 218.

17. Yoshihara K, Shahmoradgoli M, Martínez E, Vegesna R, Kim H, Torres-Garcia W*, et al.* Inferring tumour purity and stromal and immune cell admixture from expression data. *Nature communications* 2013, **4:** 2612.

18. Jeschke J, Bizet M, Desmedt C, Calonne E, Dedeurwaerder S, Garaud S*, et al.* DNA methylation-based immune response signature improves patient diagnosis in multiple cancers. *J Clin Invest* 2017, **127**(8)**:** 3090-3102.

19. Sanchez-Vega F, Mina M, Armenia J, Chatila WK, Luna A, La KC*, et al.* Oncogenic signaling pathways in the cancer genome atlas. *Cell* 2018, **173**(2)**:** 321-337. e310.

20. Dreyer SB, Upstill-Goddard R, Paulus-Hock V, Paris C, Lampraki E-M, Dray E*, et al.* Targeting DNA Damage Response and Replication Stress in Pancreatic Cancer. *Gastroenterology* 2021, **160**(1)**:** 362-377.e313.

21. Mayakonda A, Lin D-C, Assenov Y, Plass C, Koeffler HP. Maftools: efficient and comprehensive analysis of somatic variants in cancer. *Genome research* 2018, **28**(11)**:** 1747-1756.

22. Rosenthal R, McGranahan N, Herrero J, Taylor BS, Swanton C. DeconstructSigs: delineating mutational processes in single tumors distinguishes DNA repair deficiencies and patterns of carcinoma evolution. *Genome biology* 2016, **17**(1)**:** 31.

23. Mermel CH, Schumacher SE, Hill B, Meyerson ML, Beroukhim R, Getz G. GISTIC2.0 facilitates sensitive and confident localization of the targets of focal somatic copy-number alteration in human cancers. *Genome Biol* 2011, **12**(4)**:** R41.

24. Tian Y, Morris TJ, Webster AP, Yang Z, Beck S, Feber A*, et al.* ChAMP: updated methylation analysis pipeline for Illumina BeadChips. *Bioinformatics* 2017, **33**(24)**:** 3982-3984.

25. Damrauer JS, Hoadley KA, Chism DD, Fan C, Tiganelli CJ, Wobker SE*, et al.* Intrinsic subtypes of high-grade bladder cancer reflect the hallmarks of breast cancer biology. *Proceedings of the National Academy of Sciences* 2014, **111**(8)**:** 3110-3115.

26. Kamoun A, de Reyniès A, Allory Y, Sjödahl G, Robertson AG, Seiler R*, et al.* A consensus molecular classification of muscle-invasive bladder cancer. *European urology* 2020, **77**(4)**:** 420-433.

27. Hoshida Y. Nearest template prediction: a single-sample-based flexible class prediction with confidence assessment. *PloS one* 2010, **5**(11)**:** e15543.

28. Audia JE, Campbell RM. Histone Modifications and Cancer. *Cold Spring Harb Perspect Biol* 2016, **8**(4)**:** a019521-a019521.

29. Geeleher P, Cox NJ, Huang RS. Clinical drug response can be predicted using baseline gene expression levels and in vitro drug sensitivity in cell lines. *Genome biology* 2014, **15**(3)**:** R47.

30. Geeleher P, Cox N, Huang RS. pRRophetic: an R package for prediction of clinical chemotherapeutic response from tumor gene expression levels. *PLoS One* 2014, **9**(9)**:** e107468.

31. Lu X, Jiang L, Zhang L, Zhu Y, Hu W, Wang J*, et al.* Immune Signature-Based Subtypes of Cervical Squamous Cell Carcinoma Tightly Associated with Human Papillomavirus Type 16 Expression, Molecular Features, and Clinical Outcome. *Neoplasia* 2019, **21**(6)**:** 591-601.

32. McGranahan N, Furness AJ, Rosenthal R, Ramskov S, Lyngaa R, Saini SK*, et al.* Clonal neoantigens elicit T cell immunoreactivity and sensitivity to immune checkpoint blockade. *Science* 2016, **351**(6280)**:** 1463-1469.

33. Rosenberg JE, Hoffman-Censits J, Powles T, van der Heijden MS, Balar AV, Necchi A*, et al.* Atezolizumab in patients with locally advanced and metastatic urothelial carcinoma who have progressed following treatment with platinum-based chemotherapy: a single-arm, multicentre, phase 2 trial. *Lancet* 2016, **387**(10031)**:** 1909-1920.

34. Mariathasan S, Turley SJ, Nickles D, Castiglioni A, Yuen K, Wang Y*, et al.* TGFβ attenuates tumour response to PD-L1 blockade by contributing to exclusion of T cells. *Nature* 2018, **554**(7693)**:** 544-548.

35. Cesano A. nCounter® PanCancer immune profiling panel (NanoString technologies, Inc., Seattle, WA). *Journal for immunotherapy of cancer* 2015, **3**(1)**:** 42.

36. Liang F, Zhang S, Wang Q, Li W. Treatment effects measured by restricted mean survival time in trials of immune checkpoint inhibitors for cancer. *Annals of Oncology* 2018, **29**(5)**:** 1320-1324.

37. Meng J, Lu X, Zhou Y, Zhang M, Ge Q, Zhou J*, et al.* Tumor immune microenvironment-based classifications of bladder cancer for enhancing the response rate of immunotherapy. *Molecular Therapy - Oncolytics* 2021, **20:** 410-421.

38. Ritchie ME, Phipson B, Wu D, Hu Y, Law CW, Shi W*, et al.* limma powers differential expression analyses for RNA-sequencing and microarray studies. *Nucleic acids research* 2015, **43**(7)**:** e47-e47.

39. Gu Z, Eils R, Schlesner M. Complex heatmaps reveal patterns and correlations in multidimensional genomic data. *Bioinformatics* 2016, **32**(18)**:** 2847-2849.

**SUPPLEMENTARY TABLES**

**Table S1.** Summarization of the eight MIBC data sets included in the study.

| **Data set** | **Archive** | **Platform** | **Data Type** | **No. of MIBC** |
| --- | --- | --- | --- | --- |
| TCGA-BLCA | TCGA | Illumina HiSeq 2000 RNA Sequencing | RNA-seq | 396 |
| GSE13507 | GEO | Illumina human-6 v2.0 expression beadchip | Microarray | 61 |
| GSE32548 | GEO | Illumina HumanHT-12 V3.0 expression beadchip | Microarray | 38 |
| GSE32894 | GEO | Illumina HumanHT-12 V3.0 expression beadchip | Microarray | 51 |
| GSE48075 | GEO | Illumina HumanHT-12 V3.0 expression beadchip | Microarray | 72 |
| GSE48276 | GEO | Illumina HumanHT-12 WG-DASL V4.0 R2 expression beadchip | Microarray | 64 |
| GSE31684 | GEO | Affymetrix Human Genome U133 Plus 2.0 Array [HG-U133_Plus_2] | Microarray | 74 |
| E-MTAB-1803 | ArrayExpress | Affymetrix GeneChip Human Genome U133 Plus 2.0 [HG-U133_Plus_2] | Microarray | 43 |

**Table S2.** Demographic and clinic characteristic descriptions for MIBC patients in different data sets.

| **Characteristics ^a^** | **TCGA-BLCA** | **E-MTAB-1803** | **GSE13507** | **GSE31684** | **GSE32548** | **GSE32894** | **GSE48075** | **GSE48276** |
| --- | --- | --- | --- | --- | --- | --- | --- | --- |
| Number of samples | 396 | 43 | 61 | 74 | 38 | 51 | 72 | 64 |
| Median survival time  (month) (95% CI) | 34.5  (27.4-57.3) | 54.0  (23.0-NA) | 17.1  (14.6-NA) | 19.6  (13.3-64.4) | NA ^b^  (20.6-NA) | NA  (24.2-NA) | 37.2  (18.7-82.4) | 47.5  (32.1-NA) |
| Number of Death (%) | 175 (44.2) | 22 (51.2) | 33 (54.1) | 56 (75.7) | 17 (44.7) | 23 (45.1) | 44 (61.1) | 34 (53.1) |
| Age (Years) ^c^ | 68.0 ± 10.6 | 68.7 ± 11.7 | 66.9 ± 9.9 | 69.5 ± 10.2 | 69.6 ± 10.0 | 66.0 ± 7.6 | 68.7 ± 10.3 | 66.0 ± 10.3 |
| Gender |  |  |  |  |  |  |  |  |
| Female | 103 | 7 | 13 | 20 | 10 | 13 | - | 11 |
| Male | 293 | 36 | 48 | 54 | 28 | 38 | - | 53 |
| Grade ^d^ |  |  |  |  |  |  |  |  |
| Well/moderately differentiated | 20 | 0 | 19 | 0 | 0 | 0 | - | - |
| Poorly differentiated | 373 | 43 | 42 | 74 | 38 | 50 | - | - |
| T-stage |  |  |  |  |  |  |  |  |
| T2 | 116 | 17 | 31 | 15 | 38 | 43 | 41 | 13 |
| T3 | 190 | 18 | 19 | 41 | 0 | 7 | 23 | 41 |
| T4 | 54 | 8 | 11 | 18 | 0 | 1 | 8 | 10 |
| ^a^ Sum of frequency numbers may not equal to the total sample size due to missing values | | | | | | | | |
| ^b^ Median survival time is incalculable because the mortality at the last follow-up time is less than 50% | | | | | | | | |
| ^c^ Age is represented as mean ± standard deviation  ^d^ Tumour grades recorded in different data sets that were defined as “high grade”, “G2” or “G3” were unified as “poorly differentiated”, and “low grade” or “G1” was unified as “well/moderately differentiated” | | | | | | | | |

**Table S3.** Distribution of clinicopathological features among four integrative consensus subtypes of MIBC in TCGA data set.

| **Variables (%)** | **Total of No.** | **iCS1 (n=89)** | **iCS2 (n=105)** | **iCS3 (n=115)** | **iCS4 (n=87)** | ***P* value** |
| --- | --- | --- | --- | --- | --- | --- |
| *Age* |  |  |  |  |  | 0.022 |
| <=70 | 225 (57) | 49 (55) | 51 (49) | 78 (68) | 47 (54) |  |
| >70 | 171 (43) | 40 (45) | 54 (51) | 37 (32) | 40 (46) |  |
| *Gender* |  |  |  |  |  | 0.011 |
| Female | 103 (26) | 31 (35) | 25 (24) | 19 (16) | 28 (32) |  |
| Male | 293 (74) | 58 (65) | 80 (76) | 96 (84) | 59 (68) |  |
| *pStage* |  |  |  |  |  | <0.001 |
| Stage I | 2 (1) | 0 | 0 | 2 (2) | 0 |  |
| Stage II | 127 (32) | 27 (30) | 17 (16) | 60 (53) | 23 (26) |  |
| Stage III | 135 (34) | 35 (40) | 35 (33) | 31 (27) | 34 (39) |  |
| Stage IV | 130 (33) | 27 (30) | 53 (51) | 20 (18) | 30 (35) |  |
| *TCGA subtype* |  |  |  |  |  | <0.001 |
| Basal_squamous | 139 (35) | 85 (96) | 6 (6) | 0 | 48 (56) |  |
| Luminal | 26 (7) | 0 | 20 (19) | 3 (3) | 3 (3) |  |
| Luminal_infiltrated | 73 (18) | 3 (3) | 70 (66) | 0 | 0 |  |
| Luminal_papillary | 139 (35) | 0 | 6 (6) | 111 (96) | 22 (25) |  |
| Neuronal | 19 (5) | 1 (1) | 3 (3) | 1 (1) | 14 (16) |  |
| *PAM subtype* |  |  |  |  |  | <0.001 |
| Basal | 194 (49) | 89 (100) | 45 (43) | 0 | 60 (69) |  |
| Luminal | 202 (51) | 0 | 60 (57) | 115 (100) | 27 (31) |  |
| *Lund subtype* |  |  |  |  |  | <0.001 |
| GU | 88 (22) | 0 | 38 (36) | 35 (30) | 15 (17) |  |
| Infil | 71 (18) | 26 (29) | 44 (42) | 0 | 1 (1) |  |
| SCCL | 103 (26) | 60 (68) | 5 (5) | 0 | 38 (44) |  |
| UroA | 111 (28) | 0 | 17 (16) | 79 (68) | 15 (17) |  |
| UroB | 23 (6) | 3 (3) | 1 (1) | 1 (1) | 18 (21) |  |
| *oneNN subtype* |  |  |  |  |  | <0.001 |
| Basal | 136 (34) | 79 (89) | 9 (9) | 1 (1) | 47 (54) |  |
| Luminal | 151 (38) | 0 | 32 (30) | 96 (83) | 23 (26) |  |
| p53-like | 109 (28) | 10 (11) | 64 (61) | 18 (16) | 17 (20) |  |
| *Consensus subtype* |  |  |  |  |  | <0.001 |
| Ba/Sq | 155 (39) | 84 (94) | 14 (13) | 1 (1) | 56 (64) |  |
| LumNS | 30 (8) | 0 | 26 (25) | 1 (1) | 3 (3) |  |
| LumP | 132 (33) | 0 | 23 (22) | 93 (81) | 16 (19) |  |
| LumU | 42 (11) | 0 | 16 (15) | 20 (17) | 6 (7) |  |
| NE-like | 6 (1) | 0 | 0 | 0 | 6 (7) |  |
| Stroma-rich | 31 (8) | 5 (6) | 26 (25) | 0 | 0 |  |

**Table S4.** Distribution of somatic mutations (≥3%) among four integrative consensus subtypes of MIBC in TCGA data set.

| **Gene** | **No. of mutations** | **iCS1 (n=89)** | **iCS2 (n=105)** | **iCS3 (n=115)** | **iCS4 (n=87)** | ***P* value** |
| --- | --- | --- | --- | --- | --- | --- |
| TP53 | 196 (50%) | 66 (74.2%) | 56 (53.3%) | 35 (30.4%) | 39 (44.8%) | 7.01E-09 |
| RB1 | 75 (19%) | 35 (39.3%) | 13 (12.4%) | 9 (7.8%) | 18 (20.7%) | 1.52E-07 |
| FGFR3 | 57 (14%) | 2 (2.2%) | 6 (5.7%) | 39 (33.9%) | 10 (11.5%) | 3.44E-11 |
| STAG2 | 55 (14%) | 5 (5.6%) | 11 (10.5%) | 25 (21.7%) | 14 (16.1%) | 5.43E-03 |
| AKAP9 | 45 (11%) | 13 (14.6%) | 16 (15.2%) | 13 (11.3%) | 3 (3.4%) | 2.79E-02 |
| KMT2A | 44 (11%) | 16 (18.0%) | 14 (13.3%) | 6 (5.2%) | 8 (9.2%) | 2.47E-02 |
| DIDO1 | 33 (8%) | 12 (13.5%) | 11 (10.5%) | 4 (3.5%) | 6 (6.9%) | 4.85E-02 |
| ARID2 | 32 (8%) | 5 (5.6%) | 15 (14.3%) | 9 (7.8%) | 3 (3.4%) | 4.28E-02 |
| RELN | 31 (8%) | 5 (5.6%) | 12 (11.4%) | 13 (11.3%) | 1 (1.1%) | 1.01E-02 |
| COL6A6 | 29 (7%) | 3 (3.4%) | 14 (13.3%) | 9 (7.8%) | 3 (3.4%) | 2.81E-02 |
| SPEN | 28 (7%) | 4 (4.5%) | 10 (9.5%) | 13 (11.3%) | 1 (1.1%) | 1.35E-02 |
| SMARCA2 | 26 (7%) | 5 (5.6%) | 10 (9.5%) | 11 (9.6%) | 0 (0.0%) | 6.42E-03 |
| NFE2L2 | 25 (6%) | 3 (3.4%) | 7 (6.7%) | 1 (0.9%) | 14 (16.1%) | 1.13E-04 |
| KIF21A | 23 (6%) | 9 (10.1%) | 8 (7.6%) | 5 (4.3%) | 1 (1.1%) | 4.56E-02 |
| MEGF8 | 23 (6%) | 7 (7.9%) | 5 (4.8%) | 11 (9.6%) | 0 (0.0%) | 9.07E-03 |
| TRANK1 | 23 (6%) | 0 (0.0%) | 6 (5.7%) | 8 (7.0%) | 9 (10.3%) | 9.41E-03 |
| MED12 | 22 (6%) | 1 (1.1%) | 12 (11.4%) | 5 (4.3%) | 4 (4.6%) | 1.77E-02 |
| KIAA0947 | 22 (6%) | 1 (1.1%) | 12 (11.4%) | 9 (7.8%) | 0 (0.0%) | 2.41E-04 |
| INO80 | 22 (6%) | 6 (6.7%) | 12 (11.4%) | 3 (2.6%) | 1 (1.1%) | 7.21E-03 |
| SMG1 | 21 (5%) | 3 (3.4%) | 11 (10.5%) | 6 (5.2%) | 1 (1.1%) | 3.38E-02 |
| PSIP1 | 20 (5%) | 1 (1.1%) | 7 (6.7%) | 10 (8.7%) | 2 (2.3%) | 4.47E-02 |
| CADPS | 20 (5%) | 0 (0.0%) | 10 (9.5%) | 7 (6.1%) | 3 (3.4%) | 9.96E-03 |
| C2CD3 | 20 (5%) | 3 (3.4%) | 12 (11.4%) | 3 (2.6%) | 2 (2.3%) | 1.58E-02 |
| ZMYM4 | 20 (5%) | 6 (6.7%) | 7 (6.7%) | 7 (6.1%) | 0 (0.0%) | 4.59E-02 |
| GPR158 | 19 (5%) | 6 (6.7%) | 9 (8.6%) | 4 (3.5%) | 0 (0.0%) | 1.51E-02 |
| SOX5 | 19 (5%) | 0 (0.0%) | 5 (4.8%) | 9 (7.8%) | 5 (5.7%) | 3.23E-02 |
| PHLDB2 | 19 (5%) | 3 (3.4%) | 11 (10.5%) | 3 (2.6%) | 2 (2.3%) | 3.36E-02 |
| DENND5B | 19 (5%) | 5 (5.6%) | 8 (7.6%) | 6 (5.2%) | 0 (0.0%) | 4.21E-02 |
| STON1-GTF2A1L | 19 (5%) | 3 (3.4%) | 4 (3.8%) | 11 (9.6%) | 1 (1.1%) | 4.37E-02 |
| FER1L6 | 18 (4%) | 3 (3.4%) | 1 (1.0%) | 6 (5.2%) | 8 (9.2%) | 4.81E-02 |
| NES | 18 (4%) | 2 (2.2%) | 11 (10.5%) | 2 (1.7%) | 3 (3.4%) | 1.52E-02 |
| LPHN3 | 18 (4%) | 0 (0.0%) | 9 (8.6%) | 6 (5.2%) | 3 (3.4%) | 2.13E-02 |
| METTL3 | 18 (4%) | 1 (1.1%) | 10 (9.5%) | 6 (5.2%) | 1 (1.1%) | 1.41E-02 |
| GRM5 | 18 (4%) | 1 (1.1%) | 2 (1.9%) | 10 (8.7%) | 5 (5.7%) | 3.06E-02 |
| TMEM132D | 18 (4%) | 2 (2.2%) | 10 (9.5%) | 5 (4.3%) | 1 (1.1%) | 3.49E-02 |
| DLC1 | 18 (4%) | 4 (4.5%) | 8 (7.6%) | 6 (5.2%) | 0 (0.0%) | 4.68E-02 |
| SBNO1 | 17 (4%) | 1 (1.1%) | 7 (6.7%) | 2 (1.7%) | 7 (8.0%) | 3.42E-02 |
| SSH3 | 17 (4%) | 0 (0.0%) | 7 (6.7%) | 7 (6.1%) | 3 (3.4%) | 4.86E-02 |
| TNRC6A | 17 (4%) | 1 (1.1%) | 10 (9.5%) | 4 (3.5%) | 2 (2.3%) | 2.82E-02 |
| TANC1 | 17 (4%) | 1 (1.1%) | 10 (9.5%) | 5 (4.3%) | 1 (1.1%) | 1.46E-02 |
| PPFIA2 | 16 (4%) | 2 (2.2%) | 3 (2.9%) | 10 (8.7%) | 1 (1.1%) | 4.25E-02 |
| NLRC5 | 16 (4%) | 9 (10.1%) | 2 (1.9%) | 5 (4.3%) | 0 (0.0%) | 3.43E-03 |
| AFF4 | 16 (4%) | 7 (7.9%) | 0 (0.0%) | 6 (5.2%) | 3 (3.4%) | 1.75E-02 |
| C1ORF173 | 16 (4%) | 2 (2.2%) | 7 (6.7%) | 7 (6.1%) | 0 (0.0%) | 3.51E-02 |
| NCOA3 | 16 (4%) | 2 (2.2%) | 8 (7.6%) | 6 (5.2%) | 0 (0.0%) | 2.67E-02 |
| TRIOBP | 16 (4%) | 0 (0.0%) | 9 (8.6%) | 4 (3.5%) | 3 (3.4%) | 1.92E-02 |
| SHANK1 | 16 (4%) | 1 (1.1%) | 10 (9.5%) | 3 (2.6%) | 2 (2.3%) | 2.03E-02 |
| PIK3C2A | 16 (4%) | 0 (0.0%) | 6 (5.7%) | 8 (7.0%) | 2 (2.3%) | 3.08E-02 |
| PAN2 | 15 (4%) | 0 (0.0%) | 7 (6.7%) | 2 (1.7%) | 6 (6.9%) | 1.24E-02 |
| ITGA4 | 15 (4%) | 1 (1.1%) | 10 (9.5%) | 3 (2.6%) | 1 (1.1%) | 7.99E-03 |
| ADAMTS16 | 15 (4%) | 9 (10.1%) | 3 (2.9%) | 2 (1.7%) | 1 (1.1%) | 1.01E-02 |
| RNF123 | 14 (4%) | 1 (1.1%) | 9 (8.6%) | 3 (2.6%) | 1 (1.1%) | 2.12E-02 |
| GABRA4 | 14 (4%) | 2 (2.2%) | 0 (0.0%) | 5 (4.3%) | 7 (8.0%) | 1.20E-02 |
| TMCO4 | 13 (3%) | 0 (0.0%) | 3 (2.9%) | 9 (7.8%) | 1 (1.1%) | 7.97E-03 |
| NSMAF | 13 (3%) | 3 (3.4%) | 8 (7.6%) | 0 (0.0%) | 2 (2.3%) | 9.02E-03 |
| VWA3B | 13 (3%) | 2 (2.2%) | 8 (7.6%) | 1 (0.9%) | 2 (2.3%) | 4.78E-02 |
| ADAM10 | 13 (3%) | 2 (2.2%) | 8 (7.6%) | 3 (2.6%) | 0 (0.0%) | 2.35E-02 |
| DNAJC6 | 13 (3%) | 2 (2.2%) | 3 (2.9%) | 8 (7.0%) | 0 (0.0%) | 4.37E-02 |
| SCAPER | 13 (3%) | 0 (0.0%) | 6 (5.7%) | 7 (6.1%) | 0 (0.0%) | 4.04E-03 |
| CTNNB1 | 13 (3%) | 3 (3.4%) | 1 (1.0%) | 2 (1.7%) | 7 (8.0%) | 4.66E-02 |
| CDH16 | 13 (3%) | 3 (3.4%) | 7 (6.7%) | 0 (0.0%) | 3 (3.4%) | 2.42E-02 |
| GYS2 | 13 (3%) | 0 (0.0%) | 7 (6.7%) | 5 (4.3%) | 1 (1.1%) | 2.72E-02 |
| N4BP2L2 | 13 (3%) | 1 (1.1%) | 5 (4.8%) | 1 (0.9%) | 6 (6.9%) | 4.97E-02 |
| FANCI | 13 (3%) | 1 (1.1%) | 6 (5.7%) | 6 (5.2%) | 0 (0.0%) | 3.78E-02 |
| RNF19A | 13 (3%) | 1 (1.1%) | 11 (10.5%) | 1 (0.9%) | 0 (0.0%) | 5.86E-05 |
| SHANK2 | 13 (3%) | 5 (5.6%) | 0 (0.0%) | 2 (1.7%) | 6 (6.9%) | 1.04E-02 |
| TCF20 | 12 (3%) | 2 (2.2%) | 7 (6.7%) | 0 (0.0%) | 3 (3.4%) | 1.97E-02 |
| ZNF394 | 12 (3%) | 0 (0.0%) | 6 (5.7%) | 6 (5.2%) | 0 (0.0%) | 7.89E-03 |
| LRPPRC | 12 (3%) | 2 (2.2%) | 2 (1.9%) | 8 (7.0%) | 0 (0.0%) | 2.70E-02 |
| UVSSA | 12 (3%) | 7 (7.9%) | 3 (2.9%) | 2 (1.7%) | 0 (0.0%) | 1.66E-02 |
| CD163L1 | 12 (3%) | 3 (3.4%) | 7 (6.7%) | 2 (1.7%) | 0 (0.0%) | 4.01E-02 |
| SLC38A10 | 12 (3%) | 0 (0.0%) | 7 (6.7%) | 1 (0.9%) | 4 (4.6%) | 1.03E-02 |
| GNA13 | 12 (3%) | 0 (0.0%) | 8 (7.6%) | 2 (1.7%) | 2 (2.3%) | 1.25E-02 |
| KIT | 12 (3%) | 3 (3.4%) | 7 (6.7%) | 2 (1.7%) | 0 (0.0%) | 4.01E-02 |
| TTC37 | 12 (3%) | 2 (2.2%) | 0 (0.0%) | 7 (6.1%) | 3 (3.4%) | 4.21E-02 |
| ALAS1 | 12 (3%) | 3 (3.4%) | 7 (6.7%) | 0 (0.0%) | 2 (2.3%) | 2.08E-02 |
| ZNF600 | 12 (3%) | 7 (7.9%) | 1 (1.0%) | 3 (2.6%) | 1 (1.1%) | 3.58E-02 |

**Table S5.** List of markers for predicting each integrative consensus subtypes of MIBC.

| iCS1 | iCS2 | iCS3 | iCS4 |
| --- | --- | --- | --- |
| SAA1 | MYH11 | BTBD16 | KRT4 |
| CXCL10 | PTN | SLC14A1 | SOX2 |
| CXCL11 | IGFL1 | HPGD | AKR1B10 |
| MT2A | MFAP4 | ANXA10 | CEACAM5 |
| GBP5 | HSPB6 | CRTAC1 | SCGB1A1 |
| S100A8 | LMOD1 | SHH | ALDH3A1 |
| KLK5 | PTGIS | AGR2 | ADH7 |
| IDO1 | PCP4 | SLITRK6 | SLC7A11 |
| GZMA | GABBR2 | CYP3A5 | ZIC2 |
| GZMB | PODN | SLC44A4 | MUC4 |
| TGFBI | SMOC2 | HSD17B2 | IRX3 |
| SERPINA1 | SPON1 | TFF1 | SERPINB5 |
| PDCD1LG2 | PRELP | FAM3D | CALML3 |
| NKG7 | ELN | CTSE | CEACAM6 |
| CCL13 | CASQ2 | FOXQ1 | OLFM4 |
| GNLY | KCNG1 | PHGR1 | NRARP |
| MMP12 | APOD | CLCA4 | SOX21 |
| CCL4 | TMEM119 | ERN2 | CCDC190 |
| CCL8 | C7 | EEF1A2 | PRSS12 |
| SPHK1 | GATM | FABP6 | KRT15 |
| PRF1 | CCR7 | FGFR3 | CELSR2 |
| WARS | KRT23 | PNCK | NXPH4 |
| G0S2 | PGM5 | PTPRR | DAPL1 |
| LAG3 | SYNPO2 | ATOH8 | ABCA4 |
| ZBED2 | NCCRP1 | KRTAP5-9 | HOXD13 |
| CCL3 | CLDN3 | AQP3 | TMPRSS13 |
| NT5E | AOC3 | SPINK4 | CBX2 |
| CXCL5 | MRVI1 | SIGLEC15 | MAP2 |
| PDZK1IP1 | COL14A1 | ADGRF1 | RASAL1 |
| CD274 | PLA2G2A | EPHB6 | ADAM23 |

**Table S6.** Gene list related to 24 specific microenvironment cell types.

| Symbol | Cell type | Symbol | Cell type | Symbol | Cell type | Symbol | Cell type |
| --- | --- | --- | --- | --- | --- | --- | --- |
| ABCB4 | B.cells.naive | ICOS | T.cells.follicular.helper | CCL19 | Macrophages.M1 | LRMP | Eosinophils |
| ADAM28 | B.cells.naive | IL21 | T.cells.follicular.helper | CCL8 | Macrophages.M1 | NR4A3 | Eosinophils |
| BACH2 | B.cells.naive | MAP4K1 | T.cells.follicular.helper | CD40 | Macrophages.M1 | OSM | Eosinophils |
| BCL7A | B.cells.naive | PASK | T.cells.follicular.helper | CXCL10 | Macrophages.M1 | P2RY10 | Eosinophils |
| BEND5 | B.cells.naive | PDCD1 | T.cells.follicular.helper | CXCL11 | Macrophages.M1 | P2RY14 | Eosinophils |
| BRAF | B.cells.naive | SLC7A10 | T.cells.follicular.helper | CXCL9 | Macrophages.M1 | P2RY2 | Eosinophils |
| CD22 | B.cells.naive | ST8SIA1 | T.cells.follicular.helper | CYP27B1 | Macrophages.M1 | PDE6C | Eosinophils |
| CD72 | B.cells.naive | TRIB2 | T.cells.follicular.helper | EBI3 | Macrophages.M1 | PKD2L2 | Eosinophils |
| CR2 | B.cells.naive | TSHR | T.cells.follicular.helper | HESX1 | Macrophages.M1 | RGS1 | Eosinophils |
| GPR18 | B.cells.naive | ZAP70 | T.cells.follicular.helper | MACF1 | Macrophages.M1 | RNASE2 | Eosinophils |
| HHEX | B.cells.naive | ZBTB10 | T.cells.follicular.helper | NOD2 | Macrophages.M1 | RRP12 | Eosinophils |
| IL4R | B.cells.naive | BARX2 | T.cells.regulatory..Tregs. | PLA1A | Macrophages.M1 | SAMSN1 | Eosinophils |
| ZNF263 | B.cells.naive | CD5 | T.cells.regulatory..Tregs. | SIGLEC1 | Macrophages.M1 | SMPD3 | Eosinophils |
| MEP1A | B.cells.naive | CD7 | T.cells.regulatory..Tregs. | SLAMF1 | Macrophages.M1 | SMPDL3B | Eosinophils |
| NIPSNAP3B | B.cells.naive | CD70 | T.cells.regulatory..Tregs. | SLC2A6 | Macrophages.M1 | TRPM6 | Eosinophils |
| SLC12A1 | B.cells.naive | CEMP1 | T.cells.regulatory..Tregs. | SOCS1 | Macrophages.M1 | ZNF165 | Eosinophils |
| TCL1A | B.cells.naive | CRISP3 | T.cells.regulatory..Tregs. | TLR7 | Macrophages.M1 | ZNF222 | Eosinophils |
| ZNF286A | B.cells.naive | CTLA4 | T.cells.regulatory..Tregs. | TNFAIP6 | Macrophages.M1 | AQP9 | Neutrophils |
| BLK | B.cells.memory | EFNA5 | T.cells.regulatory..Tregs. | TNIP3 | Macrophages.M1 | BTNL8 | Neutrophils |
| IL7 | B.cells.memory | FOXP3 | T.cells.regulatory..Tregs. | CCL14 | Macrophages.M2 | C5AR1 | Neutrophils |
| NPIPB15 | B.cells.memory | FRMD8 | T.cells.regulatory..Tregs. | CCL18 | Macrophages.M2 | CDA | Neutrophils |
| SP140 | B.cells.memory | HIC1 | T.cells.regulatory..Tregs. | CCL23 | Macrophages.M2 | CEACAM3 | Neutrophils |
| TRAF4 | B.cells.memory | HMGB3P30 | T.cells.regulatory..Tregs. | CD4 | Macrophages.M2 | CREB5 | Neutrophils |
| ABCB9 | Plasma.cells | KIRREL | T.cells.regulatory..Tregs. | CD68 | Macrophages.M2 | CSF3R | Neutrophils |
| AMPD1 | Plasma.cells | LAIR2 | T.cells.regulatory..Tregs. | CLEC4A | Macrophages.M2 | CXCR1 | Neutrophils |
| ANGPT4 | Plasma.cells | LILRA4 | T.cells.regulatory..Tregs. | CRYBB1 | Macrophages.M2 | CXCR2 | Neutrophils |
| ATXN8OS | Plasma.cells | LOC126987 | T.cells.regulatory..Tregs. | FRMD4A | Macrophages.M2 | FAM212B | Neutrophils |
| C11orf80 | Plasma.cells | NPAS1 | T.cells.regulatory..Tregs. | HRH1 | Macrophages.M2 | FAM65B | Neutrophils |
| CCR10 | Plasma.cells | NTN3 | T.cells.regulatory..Tregs. | MS4A6A | Macrophages.M2 | FCGR3B | Neutrophils |
| DENND5B | Plasma.cells | PLCH2 | T.cells.regulatory..Tregs. | NME8 | Macrophages.M2 | FFAR2 | Neutrophils |
| EAF2 | Plasma.cells | PMCH | T.cells.regulatory..Tregs. | NPL | Macrophages.M2 | FPR1 | Neutrophils |
| GUSBP11 | Plasma.cells | RYR1 | T.cells.regulatory..Tregs. | RENBP | Macrophages.M2 | FPR2 | Neutrophils |
| HIST1H2AE | Plasma.cells | SEC31B | T.cells.regulatory..Tregs. | WNT5B | Macrophages.M2 | HAL | Neutrophils |
| HIST1H2BG | Plasma.cells | KLHL22 | T.cells.regulatory..Tregs. | ALOX15 | Dendritic.cells.resting | HSPA6 | Neutrophils |
| IGHD | Plasma.cells | SIT1 | T.cells.regulatory..Tregs. | C1orf54 | Dendritic.cells.resting | LST1 | Neutrophils |
| IGHE | Plasma.cells | SKAP1 | T.cells.regulatory..Tregs. | CD1A | Dendritic.cells.resting | MAK | Neutrophils |
| IGLL3P | Plasma.cells | SSX1 | T.cells.regulatory..Tregs. | CD1B | Dendritic.cells.resting | MEFV | Neutrophils |
| KCNG2 | Plasma.cells | TRAV21 | T.cells.regulatory..Tregs. | CD1C | Dendritic.cells.resting | MGAM | Neutrophils |
| LOC100130100 | Plasma.cells | TRAV8-6 | T.cells.regulatory..Tregs. | CD1E | Dendritic.cells.resting | MMP25 | Neutrophils |
| MAN1A1 | Plasma.cells | TRAV9-2 | T.cells.regulatory..Tregs. | DHRS11 | Dendritic.cells.resting | MNDA | Neutrophils |
| MANEA | Plasma.cells | TYR | T.cells.regulatory..Tregs. | MMP12 | Dendritic.cells.resting | MXD1 | Neutrophils |
| MAST1 | Plasma.cells | CDH12 | T.cells.gamma.delta | PPFIBP1 | Dendritic.cells.resting | NFE2 | Neutrophils |
| MROH7 | Plasma.cells | GZMK | T.cells.gamma.delta | RNASE6 | Dendritic.cells.resting | P2RY13 | Neutrophils |
| MZB1 | Plasma.cells | TARDBPP1 | T.cells.gamma.delta | SCN9A | Dendritic.cells.resting | PGLYRP1 | Neutrophils |
| PAX7 | Plasma.cells | TRDC | T.cells.gamma.delta | TREM2 | Dendritic.cells.resting | REPS2 | Neutrophils |
| PDK1 | Plasma.cells | ZNF442 | T.cells.gamma.delta | ARHGAP22 | Dendritic.cells.activated | STEAP4 | Neutrophils |
| RASGRP3 | Plasma.cells | CDHR1 | NK.cells.resting | BIRC3 | Dendritic.cells.activated | TNFRSF10C | Neutrophils |
| REN | Plasma.cells | DEFA4 | NK.cells.resting | CCL17 | Dendritic.cells.activated | TREM1 | Neutrophils |
| SPAG4 | Plasma.cells | KLRC3 | NK.cells.resting | CCL22 | Dendritic.cells.activated | TREML2 | Neutrophils |
| ST6GALNAC4 | Plasma.cells | KLRF1 | NK.cells.resting | CD86 | Dendritic.cells.activated | VNN2 | Neutrophils |
| TGM5 | Plasma.cells | NAALADL1 | NK.cells.resting | CHST7 | Dendritic.cells.activated | VNN3 | Neutrophils |
| TNFRSF17 | Plasma.cells | S1PR5 | NK.cells.resting | CLIC2 | Dendritic.cells.activated | ACVRL1 | Endothelial cells |
| UGT2B17 | Plasma.cells | TEP1 | NK.cells.resting | ETV3 | Dendritic.cells.activated | APLN | Endothelial cells |
| CD8A | T.cells.CD8 | TTC38 | NK.cells.resting | HTR2B | Dendritic.cells.activated | BCL6B | Endothelial cells |
| CD8B | T.cells.CD8 | ZNF135 | NK.cells.resting | IL12B | Dendritic.cells.activated | BMP6 | Endothelial cells |
| CRTAM | T.cells.CD8 | CCND2 | NK.cells.activated | MAP3K13 | Dendritic.cells.activated | BMX | Endothelial cells |
| TRAV12-2 | T.cells.CD8 | CDK6 | NK.cells.activated | PDCD1LG2 | Dendritic.cells.activated | CDH5 | Endothelial cells |
| ANKRD55 | T.cells.CD4.naive | CTSW | NK.cells.activated | ADAMTS3 | Mast.cells.resting | CLEC14A | Endothelial cells |
| ATHL1 | T.cells.CD4.naive | GZMA | NK.cells.activated | ADRB2 | Mast.cells.resting | CXorf36 | Endothelial cells |
| DSC1 | T.cells.CD4.naive | IL12RB2 | NK.cells.activated | FAM124B | Mast.cells.resting | EDN1 | Endothelial cells |
| EPHA1 | T.cells.CD4.naive | KIR2DL1 | NK.cells.activated | FAM174B | Mast.cells.resting | ELTD1 | Endothelial cells |
| FLT3LG | T.cells.CD4.naive | KIR2DL4 | NK.cells.activated | GFI1 | Mast.cells.resting | EMCN | Endothelial cells |
| GAL3ST4 | T.cells.CD4.naive | KIR2DS4 | NK.cells.activated | HOXA1 | Mast.cells.resting | ESAM | Endothelial cells |
| GALR1 | T.cells.CD4.naive | KIR3DL2 | NK.cells.activated | MS4A2 | Mast.cells.resting | ESM1 | Endothelial cells |
| LEF1 | T.cells.CD4.naive | NCR3 | NK.cells.activated | GADD45B | Mast.cells.resting | HECW2 | Endothelial cells |
| MAP4K2 | T.cells.CD4.naive | TNFSF14 | NK.cells.activated | AZU1 | Mast.cells.activated | HHIP | Endothelial cells |
| UBASH3A | T.cells.CD4.naive | ASGR1 | Monocytes | CCL1 | Mast.cells.activated | KDR | Endothelial cells |
| WNT7A | T.cells.CD4.naive | ASGR2 | Monocytes | CCL20 | Mast.cells.activated | MMRN1 | Endothelial cells |
| ZNF204P | T.cells.CD4.naive | CCR2 | Monocytes | CXCL3 | Mast.cells.activated | MMRN2 | Endothelial cells |
| ZNF324 | T.cells.CD4.naive | CD1D | Monocytes | IL1B | Mast.cells.activated | MYCT1 | Endothelial cells |
| EPB41 | T.cells.CD4.memory.resting | CD33 | Monocytes | IL5 | Mast.cells.activated | PALMD | Endothelial cells |
| ETS1 | T.cells.CD4.memory.resting | CFP | Monocytes | LINC00597 | Mast.cells.activated | PEAR1 | Endothelial cells |
| FBXL8 | T.cells.CD4.memory.resting | FCN1 | Monocytes | ARVCF | Mast.cells.activated | PGF | Endothelial cells |
| RCAN3 | T.cells.CD4.memory.resting | UPK3A | Monocytes | NOX3 | Mast.cells.activated | PLXNA2 | Endothelial cells |
| RPL10L | T.cells.CD4.memory.resting | BHLHE41 | Macrophages.M0 | NTRK1 | Mast.cells.activated | PTPRB | Endothelial cells |
| TRAV13-2 | T.cells.CD4.memory.resting | CHI3L1 | Macrophages.M0 | TEC | Mast.cells.activated | ROBO4 | Endothelial cells |
| CDC25A | T.cells.CD4.memory.activated | COL8A2 | Macrophages.M0 | BCL2A1 | Eosinophils | SDPR | Endothelial cells |
| IFNG | T.cells.CD4.memory.activated | CSF1 | Macrophages.M0 | C3AR1 | Eosinophils | SHANK3 | Endothelial cells |
| IL17A | T.cells.CD4.memory.activated | CXCL5 | Macrophages.M0 | CCR3 | Eosinophils | SHE | Endothelial cells |
| IL26 | T.cells.CD4.memory.activated | CYP27A1 | Macrophages.M0 | CLC | Eosinophils | TEK | Endothelial cells |
| IL3 | T.cells.CD4.memory.activated | DCSTAMP | Macrophages.M0 | DACH1 | Eosinophils | TIE1 | Endothelial cells |
| IL4 | T.cells.CD4.memory.activated | GPC4 | Macrophages.M0 | DAPK2 | Eosinophils | VEPH1 | Endothelial cells |
| IL9 | T.cells.CD4.memory.activated | MARCO | Macrophages.M0 | DEPDC5 | Eosinophils | VWF | Endothelial cells |
| ORC1 | T.cells.CD4.memory.activated | MMP9 | Macrophages.M0 | EMR1 | Eosinophils | COL1A1 | Fibroblasts |
| RRP9 | T.cells.CD4.memory.activated | PLA2G7 | Macrophages.M0 | EMR3 | Eosinophils | COL3A1 | Fibroblasts |
| SKA1 | T.cells.CD4.memory.activated | PPBP | Macrophages.M0 | EPN2 | Eosinophils | COL6A1 | Fibroblasts |
| CHI3L2 | T.cells.follicular.helper | ACHE | Macrophages.M1 | GIPR | Eosinophils | COL6A2 | Fibroblasts |
| CXCL13 | T.cells.follicular.helper | ADAMDEC1 | Macrophages.M1 | GPR183 | Eosinophils | DCN | Fibroblasts |
| CXCR5 | T.cells.follicular.helper | APOL3 | Macrophages.M1 | GPR65 | Eosinophils | GREM1 | Fibroblasts |
| FZD3 | T.cells.follicular.helper | APOL6 | Macrophages.M1 | GPR97 | Eosinophils | PAMR1 | Fibroblasts |
| ICA1 | T.cells.follicular.helper | ARRB1 | Macrophages.M1 | IL5RA | Eosinophils | TAGLN | Fibroblasts |

**Table S7.** Significant broad identified by GISTIC2.0 for each integrative consensus subtype of MIBC in TCGA data set.

| iCS1 |  |  |  |  |  |  |  |  |  |
| --- | --- | --- | --- | --- | --- | --- | --- | --- | --- |
| Arm | # Genes | Amp Freq | Amp frequency score | Amp z-score | Amp q-value | Del Freq | Del frequency score | Del z-score | Del q-value |
| 1p | 2121 | 0.08 | 0.08 | 1.11 | 0.398 | 0 | 0 | -2.13 | 0.997 |
| 1q | 1955 | 0.17 | 0.17 | 4.38 | 0.000118 | 0.01 | 0.01 | -1.68 | 0.997 |
| 2p | 924 | 0.09 | 0.09 | -0.471 | 0.999 | 0.01 | 0.01 | -2.75 | 0.997 |
| 2q | 1556 | 0.03 | 0.04 | -1.46 | 0.999 | 0.04 | 0.05 | -1.08 | 0.997 |
| 3p | 1062 | 0.1 | 0.11 | 0.407 | 0.833 | 0.11 | 0.12 | 0.74 | 0.64 |
| 3q | 1139 | 0.19 | 0.2 | 3.22 | 0.00413 | 0.04 | 0.06 | -1.18 | 0.997 |
| 4p | 489 | 0.01 | 0.01 | -3.04 | 0.999 | 0.11 | 0.11 | -0.351 | 0.997 |
| 4q | 1049 | 0.01 | 0.01 | -2.56 | 0.999 | 0.13 | 0.14 | 1.11 | 0.521 |
| 5p | 270 | 0.45 | 0.45 | 8.72 | 0 | 0.01 | 0.02 | -2.36 | 0.997 |
| 5q | 1427 | 0.08 | 0.09 | 0.229 | 0.939 | 0.13 | 0.15 | 2.05 | 0.125 |
| 6p | 1173 | 0.16 | 0.16 | 2.19 | 0.0626 | 0.04 | 0.05 | -1.23 | 0.997 |
| 6q | 839 | 0.07 | 0.08 | -0.906 | 0.999 | 0.13 | 0.14 | 0.999 | 0.564 |
| 7p | 641 | 0.21 | 0.22 | 2.8 | 0.0142 | 0.01 | 0.01 | -2.71 | 0.997 |
| 7q | 1277 | 0.15 | 0.15 | 1.96 | 0.0808 | 0.03 | 0.04 | -1.55 | 0.997 |
| 8p | 580 | 0.06 | 0.09 | -0.79 | 0.999 | 0.36 | 0.38 | 7.25 | 7.85E-12 |
| 8q | 859 | 0.24 | 0.25 | 4.07 | 0.000298 | 0.04 | 0.06 | -1.33 | 0.997 |
| 9p | 422 | 0.12 | 0.13 | 0.137 | 0.965 | 0.08 | 0.09 | -1.04 | 0.997 |
| 9q | 1113 | 0.09 | 0.09 | -0.22 | 0.999 | 0.01 | 0.01 | -2.59 | 0.997 |
| 10p | 409 | 0.15 | 0.16 | 0.731 | 0.604 | 0.07 | 0.08 | -1.32 | 0.997 |
| 10q | 1268 | 0.03 | 0.04 | -1.64 | 0.999 | 0.11 | 0.12 | 0.82 | 0.618 |
| 11p | 862 | 0.04 | 0.05 | -1.64 | 0.999 | 0.12 | 0.13 | 0.597 | 0.715 |
| 11q | 1515 | 0.04 | 0.05 | -0.872 | 0.999 | 0.15 | 0.15 | 2.49 | 0.0618 |
| 12p | 575 | 0.18 | 0.2 | 2.02 | 0.0774 | 0.08 | 0.1 | -0.686 | 0.997 |
| 12q | 1447 | 0.04 | 0.05 | -1.04 | 0.999 | 0.11 | 0.12 | 1.17 | 0.521 |
| 13q | 654 | 0.13 | 0.15 | 0.777 | 0.604 | 0.08 | 0.09 | -0.752 | 0.997 |
| 14q | 1341 | 0.08 | 0.08 | -0.203 | 0.999 | 0.03 | 0.04 | -1.63 | 0.997 |
| 15q | 1355 | 0.01 | 0.01 | -2.26 | 0.999 | 0.16 | 0.16 | 2.4 | 0.0635 |
| 16p | 872 | 0.04 | 0.05 | -1.51 | 0.999 | 0.17 | 0.18 | 2.01 | 0.125 |
| 16q | 702 | 0.04 | 0.05 | -1.82 | 0.999 | 0.11 | 0.12 | 0.0349 | 0.997 |
| 17p | 683 | 0.03 | 0.04 | -1.96 | 0.999 | 0.2 | 0.21 | 2.65 | 0.052 |
| 17q | 1592 | 0.07 | 0.07 | -0.196 | 0.999 | 0.04 | 0.05 | -0.963 | 0.997 |
| 18p | 143 | 0.22 | 0.24 | 2.53 | 0.0275 | 0.06 | 0.07 | -1.65 | 0.997 |
| 18q | 446 | 0.06 | 0.07 | -1.52 | 0.999 | 0.18 | 0.19 | 1.71 | 0.211 |
| 19p | 995 | 0.02 | 0.02 | -2.32 | 0.999 | 0.09 | 0.09 | -0.345 | 0.997 |
| 19q | 1709 | 0.12 | 0.13 | 2.1 | 0.0698 | 0.04 | 0.05 | -0.677 | 0.997 |
| 20p | 355 | 0.26 | 0.27 | 3.71 | 0.000819 | 0.03 | 0.05 | -2.08 | 0.997 |
| 20q | 753 | 0.25 | 0.25 | 4.01 | 0.000298 | 0.01 | 0.01 | -2.55 | 0.997 |
| 21q | 509 | 0.07 | 0.08 | -1.22 | 0.999 | 0.15 | 0.16 | 0.867 | 0.618 |
| 22q | 921 | 0.07 | 0.09 | -0.527 | 0.999 | 0.22 | 0.24 | 3.97 | 0.000703 |
| iCS2 |  |  |  |  |  |  |  |  |  |
| Arm | # Genes | Amp Freq | Amp frequency score | Amp z-score | Amp q-value | Del Freq | Del frequency score | Del z-score | Del q-value |
| 1p | 2121 | 0.13 | 0.14 | 1.68 | 0.141 | 0.06 | 0.07 | -0.878 | 1 |
| 1q | 1955 | 0.19 | 0.2 | 3.12 | 0.00496 | 0.03 | 0.04 | -2.04 | 1 |
| 2p | 924 | 0.22 | 0.22 | 1.62 | 0.148 | 0.01 | 0.01 | -3.69 | 1 |
| 2q | 1556 | 0.08 | 0.08 | -1.28 | 1 | 0.08 | 0.08 | -1.28 | 1 |
| 3p | 1062 | 0.29 | 0.29 | 3.84 | 0.000487 | 0.02 | 0.03 | -3.06 | 1 |
| 3q | 1139 | 0.35 | 0.36 | 6.01 | 1.18E-08 | 0.03 | 0.04 | -2.45 | 1 |
| 4p | 489 | 0.07 | 0.08 | -2.53 | 1 | 0.18 | 0.19 | 0.145 | 1 |
| 4q | 1049 | 0.03 | 0.03 | -3.11 | 1 | 0.17 | 0.18 | 0.589 | 0.775 |
| 5p | 270 | 0.27 | 0.29 | 2.16 | 0.0675 | 0.08 | 0.1 | -2.13 | 1 |
| 5q | 1427 | 0.04 | 0.06 | -1.87 | 1 | 0.33 | 0.35 | 6.31 | 2.75E-09 |
| 6p | 1173 | 0.13 | 0.15 | 0.0662 | 1 | 0.11 | 0.13 | -0.436 | 1 |
| 6q | 839 | 0.04 | 0.05 | -2.85 | 1 | 0.23 | 0.24 | 1.88 | 0.13 |
| 7p | 641 | 0.24 | 0.25 | 1.73 | 0.135 | 0.03 | 0.04 | -3.31 | 1 |
| 7q | 1277 | 0.2 | 0.21 | 1.9 | 0.108 | 0.04 | 0.05 | -2.48 | 1 |
| 8p | 580 | 0.07 | 0.12 | -1.28 | 1 | 0.44 | 0.47 | 7.34 | 4.17E-12 |
| 8q | 859 | 0.38 | 0.41 | 6.5 | 7.78E-10 | 0.08 | 0.12 | -0.939 | 1 |
| 9p | 422 | 0.08 | 0.13 | -1.31 | 1 | 0.4 | 0.43 | 6.02 | 1.11E-08 |
| 9q | 1113 | 0.03 | 0.04 | -2.5 | 1 | 0.34 | 0.35 | 5.67 | 6.98E-08 |
| 10p | 409 | 0.29 | 0.3 | 2.72 | 0.0161 | 0.05 | 0.07 | -2.77 | 1 |
| 10q | 1268 | 0.07 | 0.08 | -1.69 | 1 | 0.16 | 0.17 | 0.876 | 0.572 |
| 11p | 862 | 0.03 | 0.04 | -2.71 | 1 | 0.35 | 0.36 | 5.33 | 3.79E-07 |
| 11q | 1515 | 0.07 | 0.08 | -1.33 | 1 | 0.17 | 0.18 | 1.65 | 0.194 |
| 12p | 575 | 0.1 | 0.11 | -1.92 | 1 | 0.1 | 0.12 | -1.7 | 1 |
| 12q | 1447 | 0.06 | 0.06 | -2.04 | 1 | 0.08 | 0.08 | -1.51 | 1 |
| 13q | 654 | 0.14 | 0.16 | -0.387 | 1 | 0.12 | 0.14 | -0.843 | 1 |
| 14q | 1341 | 0.05 | 0.07 | -1.8 | 1 | 0.29 | 0.3 | 4.69 | 7.78E-06 |
| 15q | 1355 | 0.04 | 0.05 | -2.48 | 1 | 0.17 | 0.18 | 1.19 | 0.389 |
| 16p | 872 | 0.1 | 0.11 | -1.43 | 1 | 0.13 | 0.15 | -0.481 | 1 |
| 16q | 702 | 0.11 | 0.13 | -1.1 | 1 | 0.13 | 0.15 | -0.637 | 1 |
| 17p | 683 | 0.01 | 0.02 | -3.44 | 1 | 0.37 | 0.38 | 5.3 | 3.79E-07 |
| 17q | 1592 | 0.15 | 0.17 | 1.28 | 0.259 | 0.09 | 0.1 | -0.643 | 1 |
| 18p | 143 | 0.17 | 0.21 | 0.0764 | 1 | 0.19 | 0.23 | 0.493 | 0.808 |
| 18q | 446 | 0.03 | 0.04 | -3.29 | 1 | 0.3 | 0.3 | 2.91 | 0.00876 |
| 19p | 995 | 0.04 | 0.04 | -3.05 | 1 | 0.11 | 0.12 | -1.09 | 1 |
| 19q | 1709 | 0.23 | 0.24 | 3.73 | 0.000623 | 0.03 | 0.04 | -2.23 | 1 |
| 20p | 355 | 0.4 | 0.43 | 5.8 | 3.23E-08 | 0.07 | 0.11 | -1.7 | 1 |
| 20q | 753 | 0.45 | 0.47 | 7.78 | 1.39E-13 | 0.04 | 0.07 | -2.09 | 1 |
| 21q | 509 | 0.25 | 0.26 | 1.87 | 0.108 | 0.05 | 0.06 | -2.82 | 1 |
| 22q | 921 | 0.03 | 0.04 | -3.16 | 1 | 0.2 | 0.21 | 1.18 | 0.389 |
| iCS3 |  |  |  |  |  |  |  |  |  |
| Arm | # Genes | Amp Freq | Amp frequency score | Amp z-score | Amp q-value | Del Freq | Del frequency score | Del z-score | Del q-value |
| 1p | 2121 | 0.09 | 0.09 | -0.533 | 1 | 0.04 | 0.05 | -1.96 | 1 |
| 1q | 1955 | 0.24 | 0.25 | 4.51 | 4.17E-05 | 0.03 | 0.05 | -2.03 | 1 |
| 2p | 924 | 0.13 | 0.14 | -0.745 | 1 | 0.09 | 0.1 | -1.87 | 1 |
| 2q | 1556 | 0.06 | 0.08 | -1.72 | 1 | 0.19 | 0.2 | 2.03 | 0.0751 |
| 3p | 1062 | 0.26 | 0.27 | 3.06 | 0.00472 | 0.03 | 0.05 | -2.89 | 1 |
| 3q | 1139 | 0.28 | 0.29 | 3.68 | 0.000769 | 0.03 | 0.04 | -3.06 | 1 |
| 4p | 489 | 0.1 | 0.12 | -1.9 | 1 | 0.12 | 0.14 | -1.47 | 1 |
| 4q | 1049 | 0.05 | 0.06 | -2.68 | 1 | 0.17 | 0.17 | 0.304 | 0.99 |
| 5p | 270 | 0.34 | 0.36 | 4.02 | 0.000226 | 0.06 | 0.09 | -2.44 | 1 |
| 5q | 1427 | 0.06 | 0.09 | -1.43 | 1 | 0.3 | 0.32 | 5.35 | 4.19E-07 |
| 6p | 1173 | 0.14 | 0.16 | 0.127 | 1 | 0.14 | 0.16 | 0.127 | 1 |
| 6q | 839 | 0.05 | 0.08 | -2.31 | 1 | 0.31 | 0.33 | 4.28 | 5.26E-05 |
| 7p | 641 | 0.23 | 0.23 | 1.33 | 0.326 | 0.03 | 0.04 | -3.41 | 1 |
| 7q | 1277 | 0.21 | 0.22 | 1.95 | 0.0991 | 0.04 | 0.05 | -2.57 | 1 |
| 8p | 580 | 0.1 | 0.13 | -1.26 | 1 | 0.29 | 0.32 | 3.36 | 0.0019 |
| 8q | 859 | 0.39 | 0.4 | 6.3 | 2.85E-09 | 0.02 | 0.03 | -3.2 | 1 |
| 9p | 422 | 0.09 | 0.18 | -0.344 | 1 | 0.51 | 0.56 | 9.41 | 0 |
| 9q | 1113 | 0.03 | 0.08 | -1.68 | 1 | 0.54 | 0.56 | 11.4 | 0 |
| 10p | 409 | 0.19 | 0.22 | 0.512 | 0.848 | 0.12 | 0.15 | -1.14 | 1 |
| 10q | 1268 | 0.07 | 0.1 | -1.29 | 1 | 0.3 | 0.33 | 5.05 | 1.73E-06 |
| 11p | 862 | 0 | 0 | -3.69 | 1 | 0.43 | 0.43 | 7.4 | 8.85E-13 |
| 11q | 1515 | 0.01 | 0.01 | -3.46 | 1 | 0.23 | 0.24 | 3.03 | 0.00533 |
| 12p | 575 | 0.17 | 0.19 | -0.00128 | 1 | 0.08 | 0.09 | -2.34 | 1 |
| 12q | 1447 | 0.14 | 0.15 | 0.293 | 1 | 0.09 | 0.1 | -1.18 | 1 |
| 13q | 654 | 0.08 | 0.09 | -2.47 | 1 | 0.12 | 0.13 | -1.39 | 1 |
| 14q | 1341 | 0.03 | 0.03 | -3.12 | 1 | 0.2 | 0.21 | 1.71 | 0.142 |
| 15q | 1355 | 0.05 | 0.06 | -2.43 | 1 | 0.14 | 0.15 | -0.0148 | 1 |
| 16p | 872 | 0.05 | 0.06 | -2.8 | 1 | 0.18 | 0.19 | 0.544 | 0.817 |
| 16q | 702 | 0.08 | 0.1 | -2.09 | 1 | 0.2 | 0.22 | 0.934 | 0.525 |
| 17p | 683 | 0.1 | 0.14 | -0.959 | 1 | 0.32 | 0.36 | 4.56 | 1.69E-05 |
| 17q | 1592 | 0.23 | 0.25 | 3.46 | 0.00134 | 0.05 | 0.07 | -1.83 | 1 |
| 18p | 143 | 0.18 | 0.22 | 0.17 | 1 | 0.17 | 0.2 | -0.227 | 1 |
| 18q | 446 | 0.09 | 0.12 | -1.72 | 1 | 0.28 | 0.3 | 2.82 | 0.00935 |
| 19p | 995 | 0.18 | 0.19 | 0.644 | 0.822 | 0.03 | 0.04 | -3.22 | 1 |
| 19q | 1709 | 0.23 | 0.24 | 3.51 | 0.00125 | 0.02 | 0.02 | -2.96 | 1 |
| 20p | 355 | 0.34 | 0.36 | 4.1 | 0.000197 | 0.05 | 0.08 | -2.65 | 1 |
| 20q | 753 | 0.45 | 0.46 | 7.78 | 1.34E-13 | 0.02 | 0.03 | -3.05 | 1 |
| 21q | 509 | 0.19 | 0.22 | 0.601 | 0.822 | 0.11 | 0.14 | -1.28 | 1 |
| 22q | 921 | 0.13 | 0.15 | -0.455 | 1 | 0.15 | 0.17 | -0.00921 | 1 |
| iCS4 |  |  |  |  |  |  |  |  |  |
| Arm | # Genes | Amp Freq | Amp frequency score | Amp z-score | Amp q-value | Del Freq | Del frequency score | Del z-score | Del q-value |
| 1p | 2121 | 0.17 | 0.18 | 1.59 | 0.181 | 0.02 | 0.03 | -2.41 | 1 |
| 1q | 1955 | 0.3 | 0.3 | 4.8 | 7.64E-06 | 0.01 | 0.02 | -2.62 | 1 |
| 2p | 924 | 0.18 | 0.19 | 0.257 | 0.905 | 0.05 | 0.06 | -2.74 | 1 |
| 2q | 1556 | 0.06 | 0.07 | -2.04 | 1 | 0.13 | 0.13 | -0.387 | 1 |
| 3p | 1062 | 0.24 | 0.27 | 2.26 | 0.0419 | 0.11 | 0.15 | -0.498 | 1 |
| 3q | 1139 | 0.43 | 0.44 | 6.48 | 1.81E-09 | 0.02 | 0.04 | -2.46 | 1 |
| 4p | 489 | 0.02 | 0.03 | -3.32 | 1 | 0.3 | 0.31 | 2.32 | 0.0562 |
| 4q | 1049 | 0.02 | 0.03 | -3.1 | 1 | 0.24 | 0.25 | 1.74 | 0.167 |
| 5p | 270 | 0.34 | 0.38 | 3.47 | 0.00102 | 0.08 | 0.12 | -1.7 | 1 |
| 5q | 1427 | 0.1 | 0.13 | -0.538 | 1 | 0.22 | 0.24 | 2.13 | 0.0805 |
| 6p | 1173 | 0.1 | 0.12 | -1.27 | 1 | 0.1 | 0.12 | -1.27 | 1 |
| 6q | 839 | 0.02 | 0.03 | -3.29 | 1 | 0.23 | 0.24 | 1.16 | 0.436 |
| 7p | 641 | 0.34 | 0.37 | 3.94 | 0.000266 | 0.07 | 0.11 | -1.73 | 1 |
| 7q | 1277 | 0.3 | 0.32 | 3.82 | 0.000365 | 0.07 | 0.1 | -1.38 | 1 |
| 8p | 580 | 0.18 | 0.25 | 1.08 | 0.391 | 0.28 | 0.34 | 2.92 | 0.0172 |
| 8q | 859 | 0.39 | 0.41 | 5.26 | 9.26E-07 | 0.05 | 0.08 | -2.06 | 1 |
| 9p | 422 | 0.07 | 0.11 | -1.91 | 1 | 0.34 | 0.37 | 3.61 | 0.00301 |
| 9q | 1113 | 0.14 | 0.18 | 0.208 | 0.905 | 0.24 | 0.28 | 2.47 | 0.0435 |
| 10p | 409 | 0.18 | 0.22 | 0.227 | 0.905 | 0.16 | 0.2 | -0.233 | 1 |
| 10q | 1268 | 0.06 | 0.08 | -1.9 | 1 | 0.25 | 0.27 | 2.54 | 0.0427 |
| 11p | 862 | 0.02 | 0.03 | -2.99 | 1 | 0.32 | 0.33 | 3.43 | 0.00398 |
| 11q | 1515 | 0.06 | 0.07 | -1.83 | 1 | 0.21 | 0.22 | 1.72 | 0.167 |
| 12p | 575 | 0.22 | 0.22 | 0.488 | 0.813 | 0.01 | 0.01 | -3.82 | 1 |
| 12q | 1447 | 0.14 | 0.14 | -0.396 | 1 | 0.01 | 0.01 | -3.39 | 1 |
| 13q | 654 | 0.14 | 0.17 | -0.458 | 1 | 0.21 | 0.24 | 0.965 | 0.544 |
| 14q | 1341 | 0.08 | 0.09 | -1.54 | 1 | 0.15 | 0.16 | 0.0503 | 1 |
| 15q | 1355 | 0.01 | 0.01 | -3.35 | 1 | 0.18 | 0.19 | 0.667 | 0.704 |
| 16p | 872 | 0.09 | 0.11 | -1.65 | 1 | 0.16 | 0.18 | -0.169 | 1 |
| 16q | 702 | 0.11 | 0.14 | -1.27 | 1 | 0.15 | 0.17 | -0.544 | 1 |
| 17p | 683 | 0.08 | 0.12 | -1.31 | 1 | 0.36 | 0.39 | 4.37 | 0.000245 |
| 17q | 1592 | 0.31 | 0.33 | 4.64 | 1.35E-05 | 0.06 | 0.08 | -1.4 | 1 |
| 18p | 143 | 0.24 | 0.28 | 1.21 | 0.342 | 0.14 | 0.18 | -0.787 | 1 |
| 18q | 446 | 0.11 | 0.14 | -1.37 | 1 | 0.18 | 0.21 | 0.0279 | 1 |
| 19p | 995 | 0.14 | 0.17 | -0.257 | 1 | 0.17 | 0.2 | 0.492 | 0.809 |
| 19q | 1709 | 0.26 | 0.28 | 3.68 | 0.000572 | 0.07 | 0.09 | -1.1 | 1 |
| 20p | 355 | 0.36 | 0.37 | 3.62 | 0.000631 | 0.05 | 0.07 | -2.56 | 1 |
| 20q | 753 | 0.41 | 0.42 | 5.38 | 7.27E-07 | 0.01 | 0.02 | -3.11 | 1 |
| 21q | 509 | 0.15 | 0.17 | -0.751 | 1 | 0.11 | 0.14 | -1.46 | 1 |
| 22q | 921 | 0.06 | 0.07 | -2.36 | 1 | 0.21 | 0.22 | 0.879 | 0.569 |

**Table S8.** Therapeutic recommendations for identified iCSs of MIBC regarding corresponding molecular characterizations.

| iCS | Molecular characterizations | Therapeutic recommendations |
| --- | --- | --- |
| Basal-inflamed | Immune-hot; Activated cell cycle pathway; activated angiogenesis pathway | immune checkpoint inhibitors; *ATR* and WEE1 inhibitors; *EGFR* inhibitors; cisplatin resistance |
| Basal-noninflamed | Immune-cold; *NRF2* mutated and activated *NRF2* oncogenic pathway; Activated cell cycle pathway | *COX* inhibitors + immune checkpoint inhibitors; *ATR* and WEE1 inhibitors; cisplatin resistance |
| Luminal-excluded | Immune hot but high genomic instability and low expression of checkpoint genes; Activated *WNT* signalling and angiogenesis pathway | ~~immune checkpoint inhibitors;~~  *WNT* inhibitors; *EGFR* inhibitors |
| Luminal-desert | Immune cold; *FGFR3* mutated | *COX* inhibitors; *FGFR3* inhibitors |

**Table S9.** Comparison of AUC between random forest and decision tree.

| AUC | Random forest | Decision tree | Difference |
| --- | --- | --- | --- |
| TCGA | 100% | 92.7% | 7.3% |
| ILLUMINA | 90.8% | 82.2% | 8.6% |
| AFFY | 85.3% | 66.4% | 18.9% |
| IMvigor210 | 92.0% | 86.3% | 5.7% |
| Mean ± SD | 92.03 ± 6.06% | 81.9 ± 11.2% | 10.1 ± 6.0% |

**Table S10.** Comparison of accuracy between random forest and decision tree.

| Accuracy | Random forest | Decision tree | Difference |
| --- | --- | --- | --- |
| TCGA | 100% | 89.8% | 10.2% |
| ILLUMINA | 83.9% | 68.6% | 15.3% |
| AFFY | 63.8% | 46.4% | 17.4% |
| IMvigor210 | 82.9% | 77.5% | 5.4% |
| Mean ± SD | 82.65 ± 14.8% | 57.18 ± 33.8% | 12.08 ± 5.4% |

**SUPPLEMENTARY FIGURES**


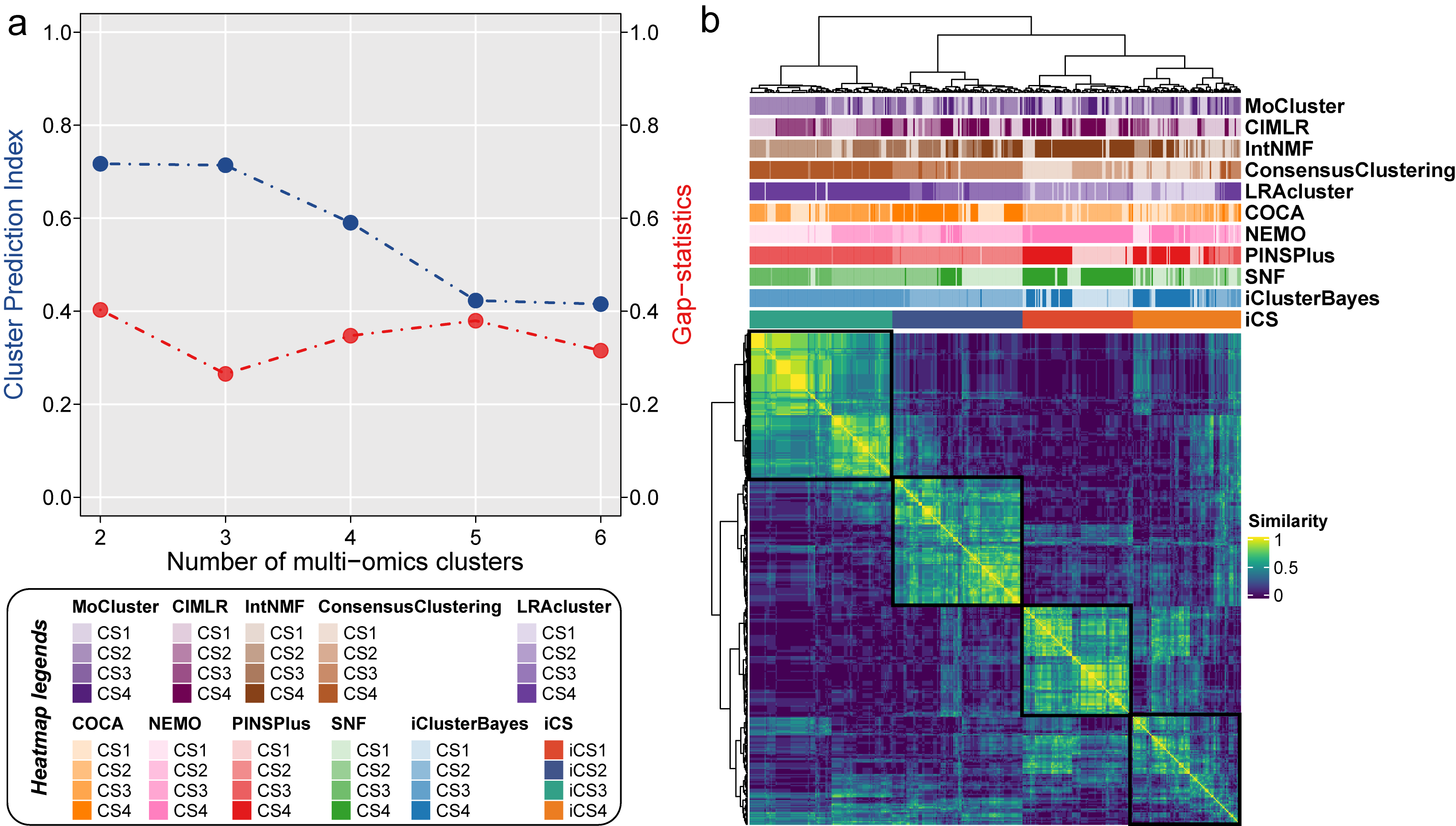


**Figure S1.** a) Identification of optimal clustering number by calculating clustering prediction index (CPI) and Gaps-statistics. b) Consensus ensemble of clustering results derived from different multi-omics integrative clustering analyses.


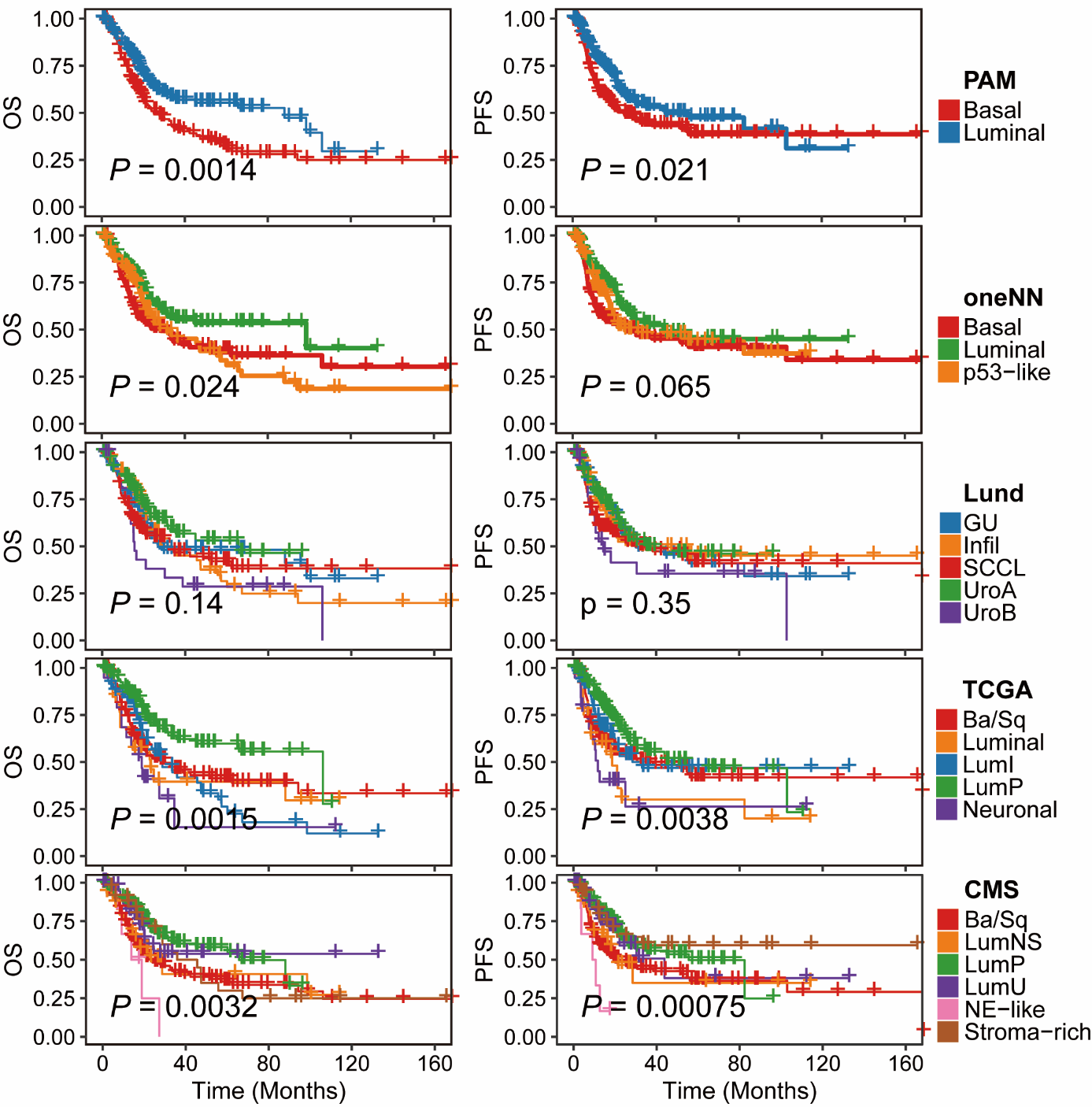


**Figure S2.** Kaplan-Meier curves of overall survival (OS) and progression-free survival (PFS) regarding prior classifications of muscle-invasive bladder cancer in MIBC-TCGA cohort. Statistical *P* values were calculated by log-rank test.

**
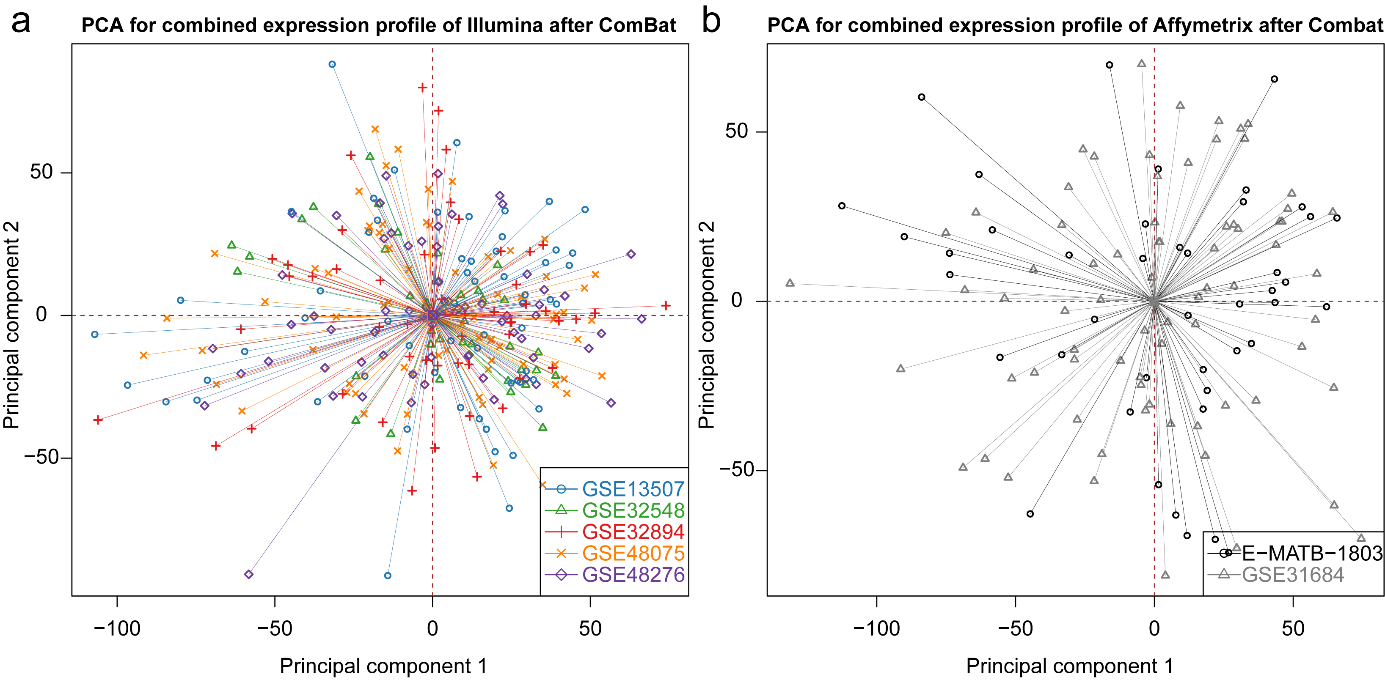
**

**Figure S3.** Principal component analysis to investigate the removal of potential batch effect across different data sets from the same sequencing platform, including a) for Illumina and b) for Affymetrix.


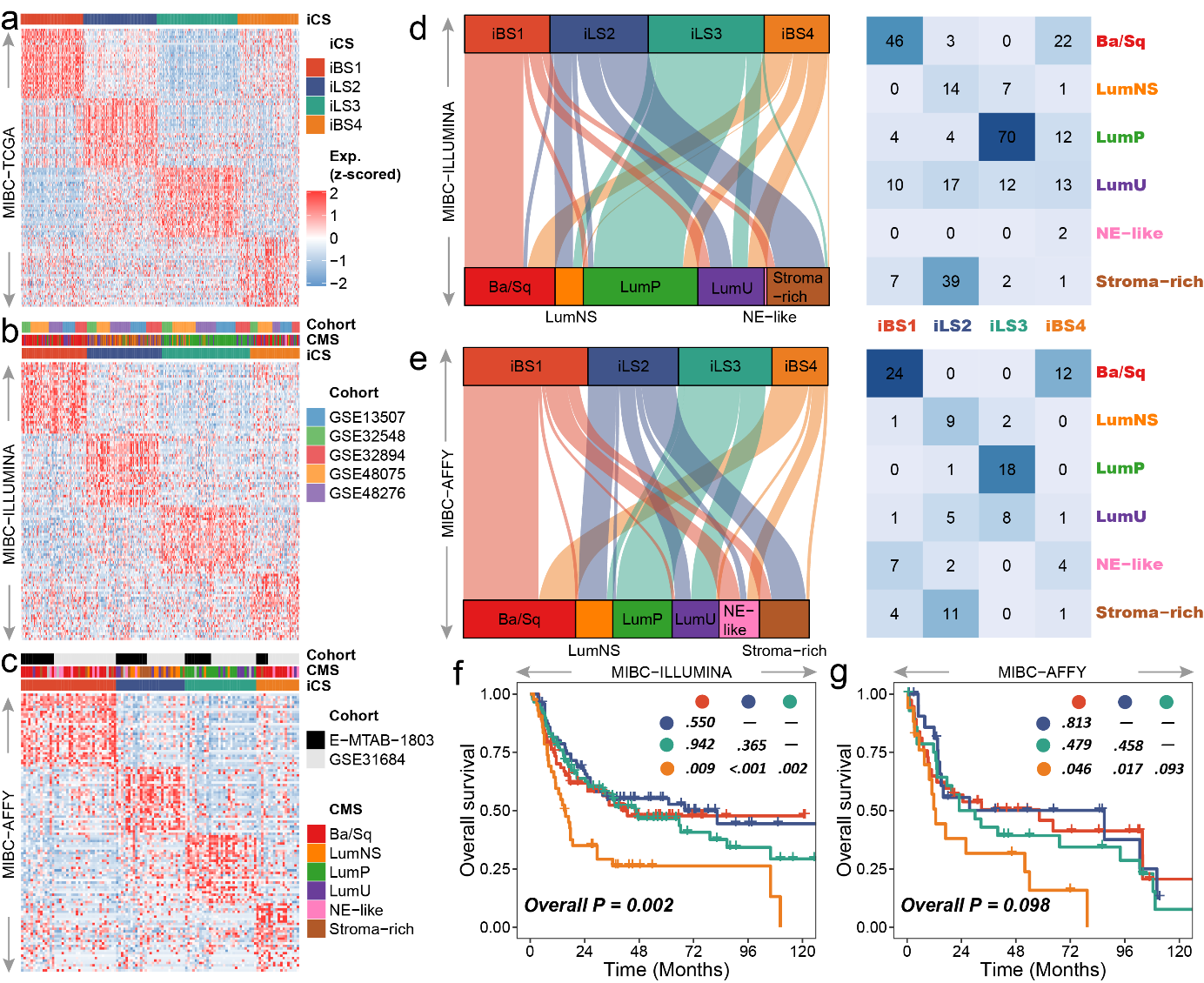


**Figure S4. Validation of the 120-gene signature to reproduce four MIBC iCS in external cohorts.** Heatmap showing the transcriptome expression pattern of the 120-gene signature in nearest template predicted iCS of three MIBC cohorts, including a) MIBC-TCGA, b) MIBC-ILLUMINA and c) MIBC-AFFY. Overlap between iCS and consensus molecular subtype (CMS) is represented in d) for MIBC-ILLUMINA and e) for MIBC-AFFY. Several patients in the MIBC-AFFY cohort had no predicted CMS because the samples showed Pearson's correlation between the gene expression profile and consensus centroid profiles lower than the confidence minimal threshold of 0.1. Kaplan-Meier curves of overall survival with the log-rank test for MIBC patients stratified by iCS are shown in f) for MIBC-ILLUMINA and g) for MIBC-AFFY.


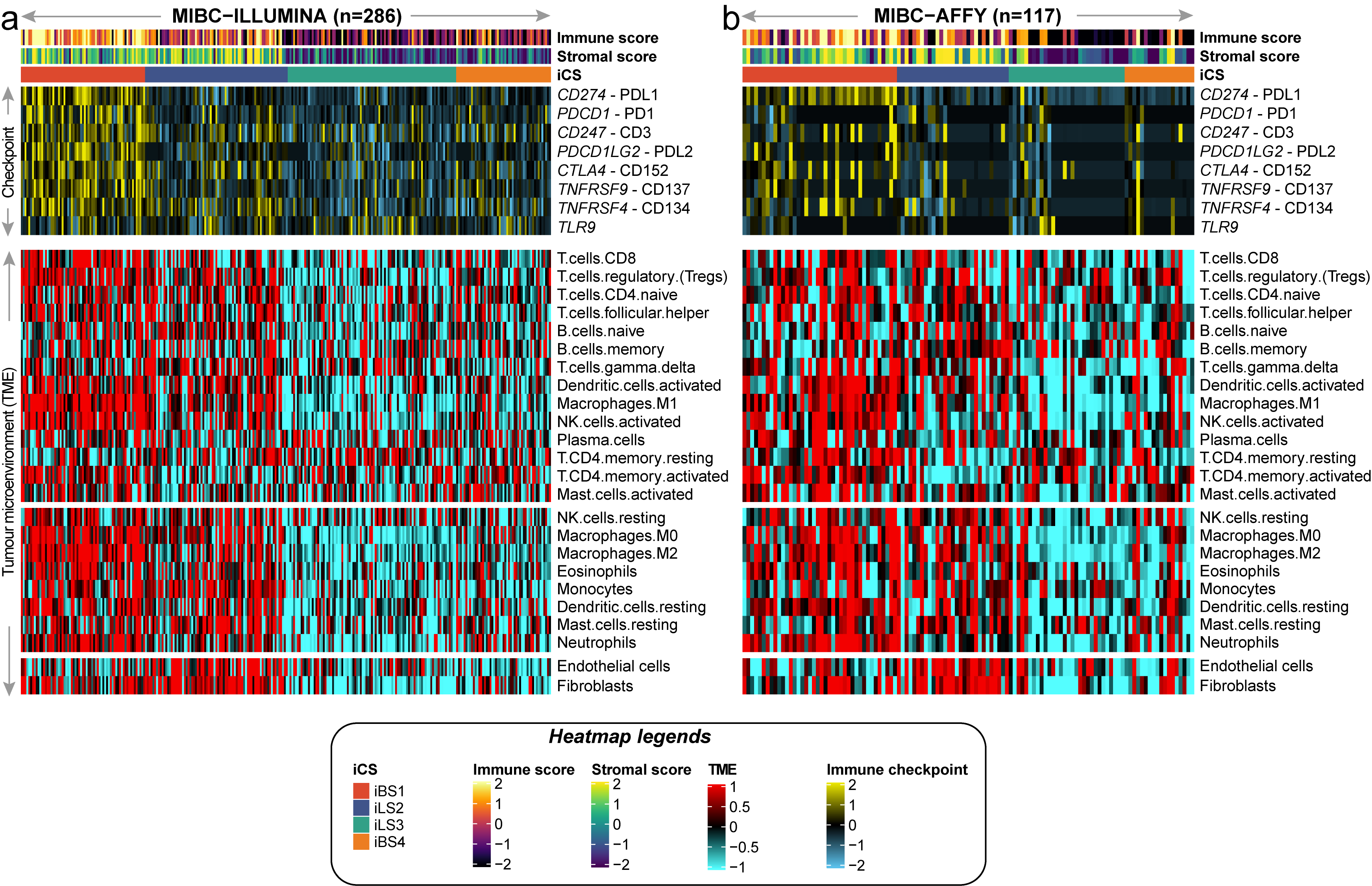


**Figure S5.** Heatmap showing immune profile in a) MIBC-ILLUMINA and b) MIBC-AFFY cohorts with the top panel for the expression of genes involved in immune checkpoint targets and the bottom panel for enrichment level of 24 microenvironment cell types. Immune enrichment score and stromal enrichment score were annotated at the top of the heatmap.


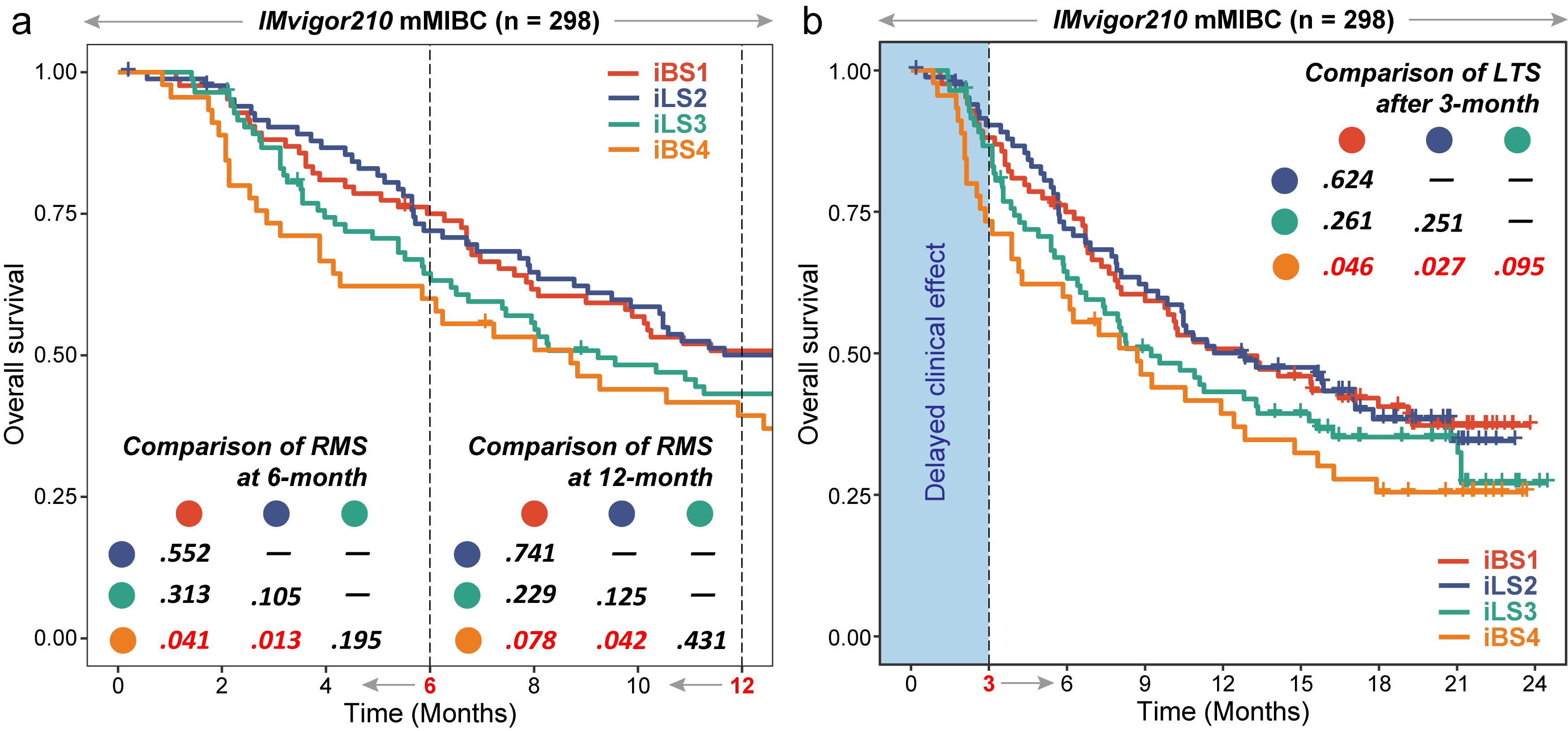


**Figure S6.** Kaplan-Meier estimator showing the overall survival curves across four iCS in IMvigor210 cohort and two non-proportional hazards statistical approaches were harnessed to compare prognosis of different iCSs. a) The restricted mean survival (RMS) time difference by six month and by one year after treatment were compared. b) The first three months after immunotherapy were considered having delayed clinical effect, thus the long-term survival after three months of treatment was compared using Chi-square(Qua) approach.


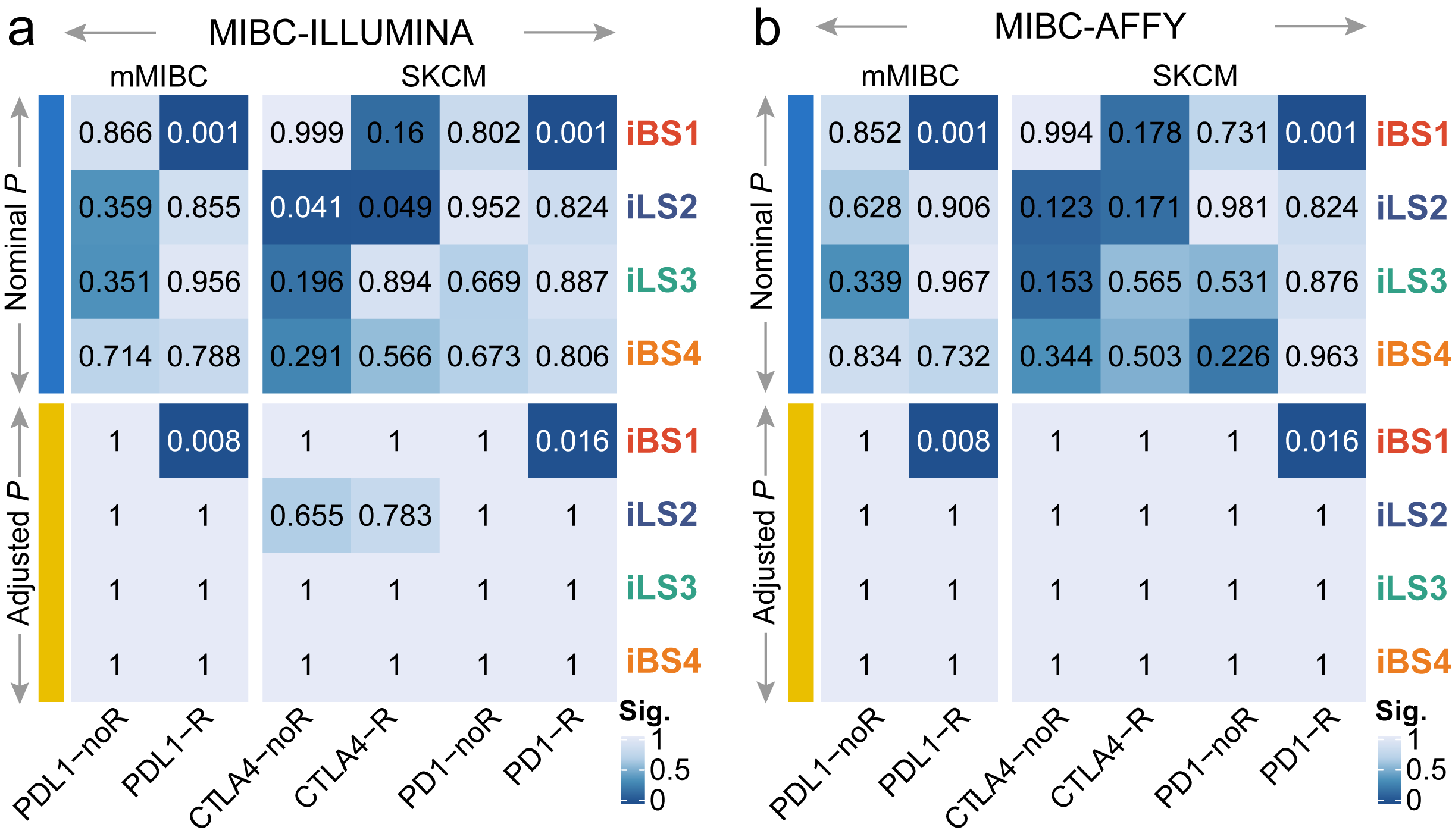


**Figure S7.** SubMap analysis manifested that iBS1 subtypes in both a) MIBC-ILLUMINA and b) MIBC-AFFY cohorts could be more sensitive to the anti-PD-L1 and anti-PD1 agents (both, Bonferroni-corrected *P* = 0.001) using two reference cohorts in which patients received immunotherapy.


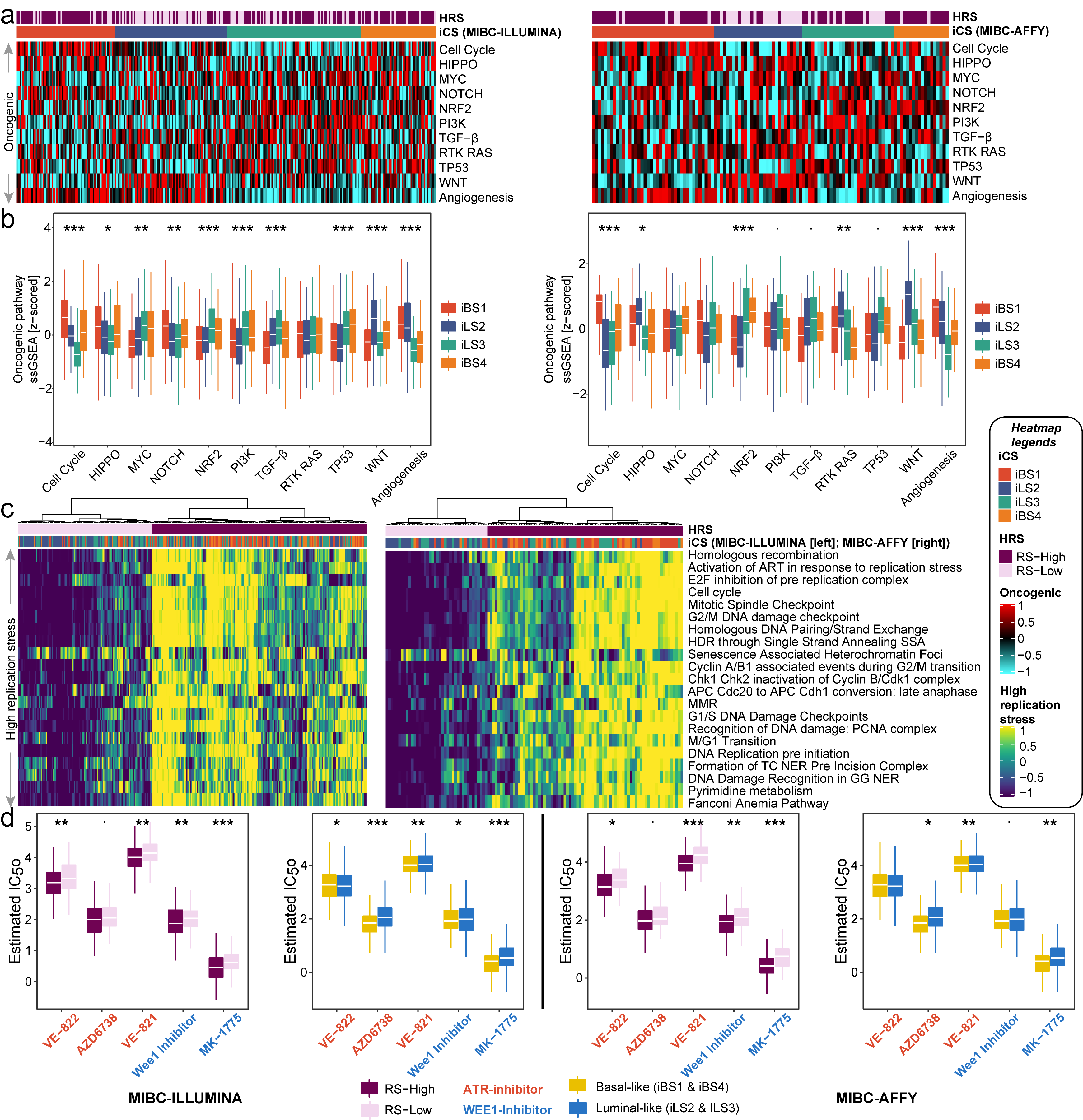


**Figure S8.** Validation of dysfunctional oncogenic pathways in MIBC-ILLUMINA (left panel) and MIBC-AFFY (right panel) cohorts. Pathway enrichment scores quantified by single-sample gene set enrichment analysis were presented in a) heatmap and b) boxplot; statistical *P* values were calculated by Kruskal-Wallis rank sum test for multiple comparison. c) Heatmap of pathways and molecular processes (Reactome database) involved in DNA maintenance and cell cycle regulation activated in replication stress and DNA damage response. Two replication stress (RS) subtypes were identified for both validation cohorts. Subtype with d) high replication stress (RS-High) or basal-like MIBC (iBS and iBS4) was inferred to be much more sensitive to both ATR (*i.e.*, VE-822, AZD6739 and VE-821) and WEE1 (*i.e.*, Wee1 inhibitor and MK-1775) inhibitors by applying a ridge regression model using 727 human cancer cell lines; statistical *P* values were calculated by two-sample Mann-Whitney U test. Drug sensitivity was measured as ln(IC_50_), and the lower the value was the more sensitive the patient would be to the treatment.


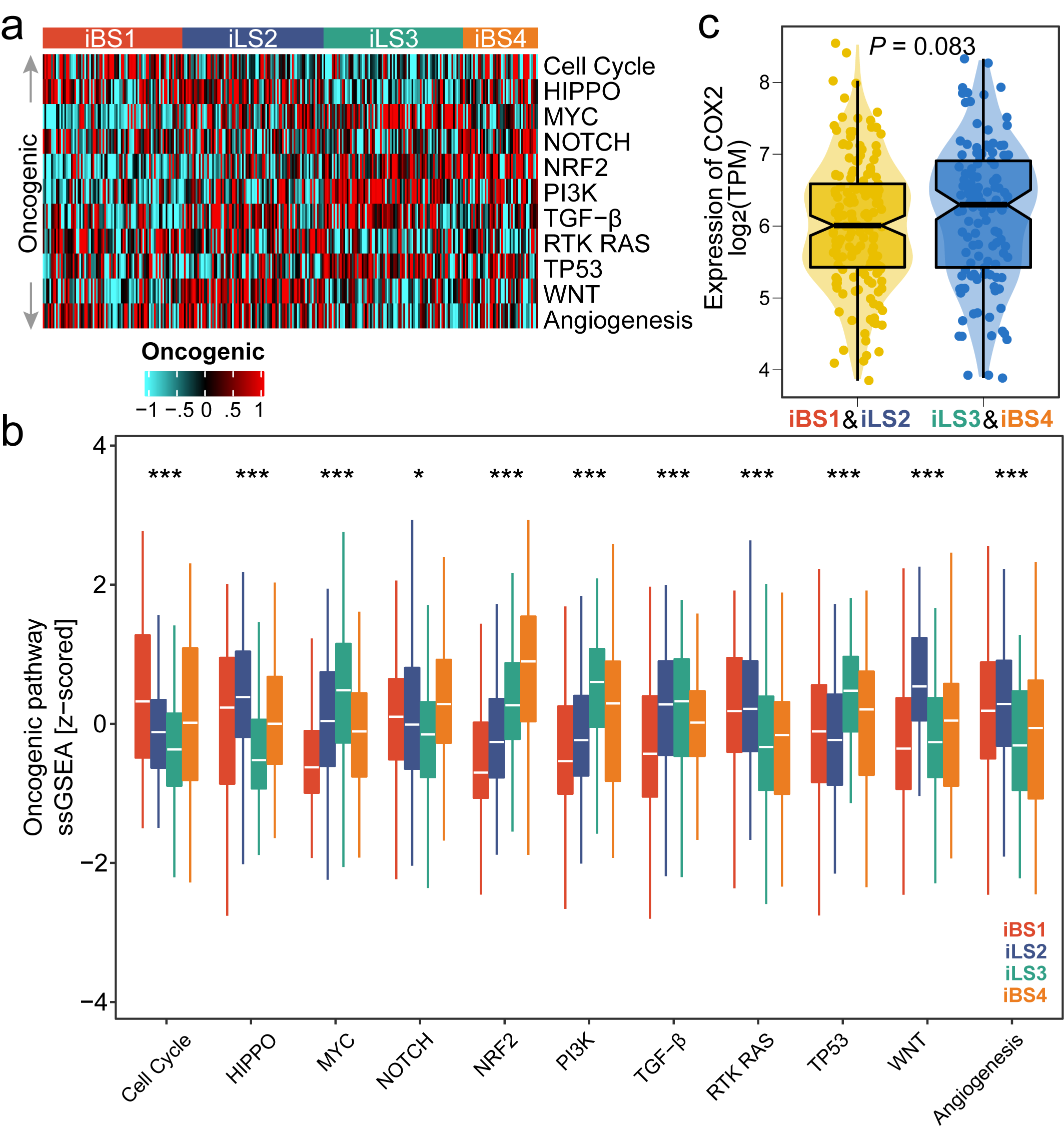


**Figure S9.** Validation of dysfunctional oncogenic pathways in IMvigor210 cohort. Dysfunctional oncogenic pathways quantified by single-sample gene set enrichment analysis were presented in a) heatmap and b) boxplot; statistical *P* values were calculated by Kruskal-Wallis rank sum test for multiple comparison. c) Distribution of *COX2* expression between immune-hot (*i.e.*, iBS1 and iLS2) and immune-cold (*i.e.*, iLS2 and iBS4) phenotype of IMvigor210 cohort; statistical *P* values were calculated by two-sample Mann-Whitney U test.


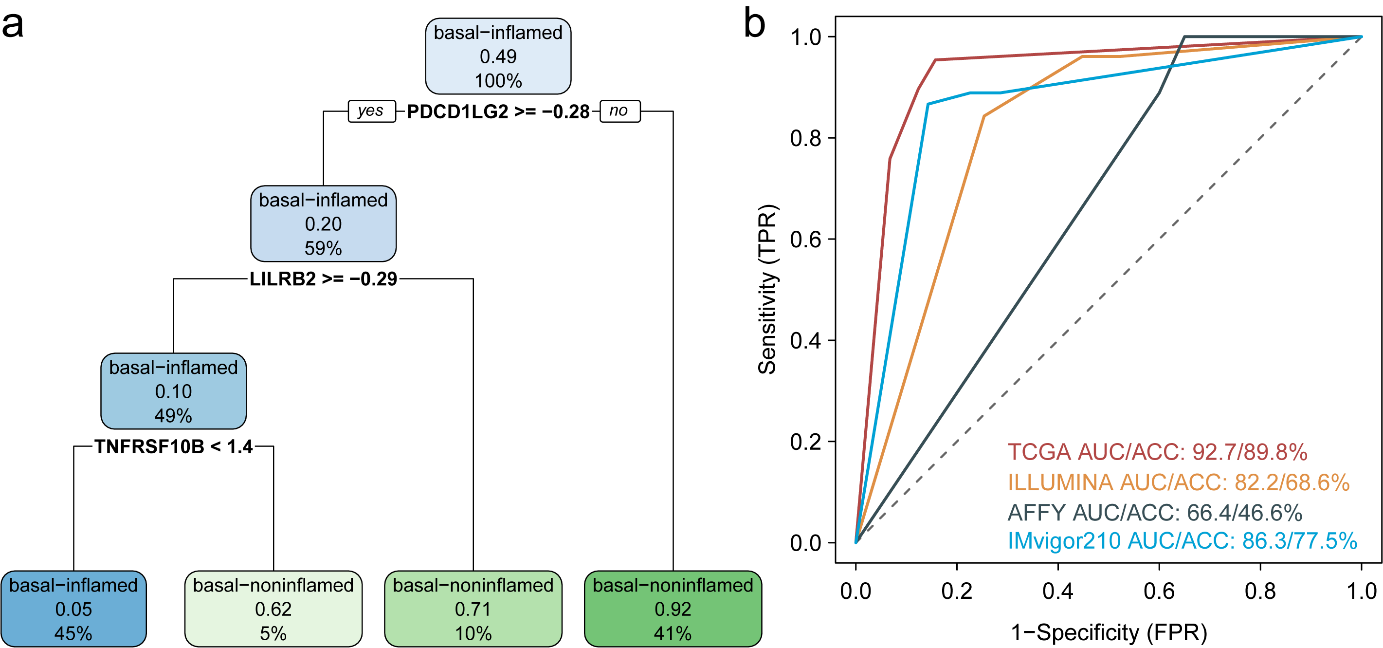


**Figure S10.** a) Development of a decision tree model (basal-tree) to refine basal-inflamed and basal-noninflamed subtypes in MIBC-TCGA cohort. b) ROC curve showing predictive performance (area under the curve [AUC] and accuracy [ACC]) when using the basal-tree to refine basal-like MIBC into basal-inflamed and basal-noninflamed subtypes.
